# The Complex Evolutionary Dynamics of Hsp70s: A Genomic and Functional Perspective

**DOI:** 10.1093/gbe/evt192

**Published:** 2013-11-24

**Authors:** Jacek Kominek, Jaroslaw Marszalek, Cécile Neuvéglise, Elizabeth A. Craig, Barry L. Williams

**Affiliations:** ^1^Laboratory of Evolutionary Biochemistry, Intercollegiate Faculty of Biotechnology, University of Gdansk, Kladki, Poland; ^2^Department of Biochemistry, University of Wisconsin–Madison; ^3^INRA Micalis UMR1319, Biologie Intégrative du Métabolisme Lipidique Microbien, Thiverval-Grignon, France; ^4^Department of Zoology, Michigan State University; ^5^Department of Microbiology and Molecular Genetics, Michigan State University

**Keywords:** molecular chaperones, multi gene family, gene duplication, birth-and-death, concerted evolution, Ascomycota genomes

## Abstract

Hsp70 molecular chaperones are ubiquitous. By preventing aggregation, promoting folding, and regulating degradation, Hsp70s are major factors in the ability of cells to maintain proteostasis. Despite a wealth of functional information, little is understood about the evolutionary dynamics of Hsp70s. We undertook an analysis of Hsp70s in the fungal clade Ascomycota. Using the well-characterized 14 Hsp70s of *Saccharomyces cerevisiae*, we identified 491 orthologs from 53 genomes. *Saccharomyces cerevisiae* Hsp70s fall into seven subfamilies: four canonical-type Hsp70 chaperones (SSA, SSB, KAR, and SSC) and three atypical Hsp70s (SSE, SSZ, and LHS) that play regulatory roles, modulating the activity of canonical Hsp70 partners. Each of the 53 surveyed genomes harbored at least one member of each subfamily, and thus establishing these seven Hsp70s as units of function and evolution. Genomes of some species contained only one member of each subfamily that is only seven Hsp70s. Overall, members of each subfamily formed a monophyletic group, suggesting that each diversified from their corresponding ancestral gene present in the common ancestor of all surveyed species. However, the pattern of evolution varied across subfamilies. At one extreme, members of the SSB subfamily evolved under concerted evolution. At the other extreme, SSA and SSC subfamilies exhibited a high degree of copy number dynamics, consistent with a birth–death mode of evolution. KAR, SSE, SSZ, and LHS subfamilies evolved in a simple divergent mode with little copy number dynamics. Together, our data revealed that the evolutionary history of this highly conserved and ubiquitous protein family was surprising complex and dynamic.

## Introduction

Hsp70s, ancient and taxonomically ubiquitous molecular chaperones, form a protein family whose members function in all major cellular compartments, participating in an extensive array of cellular processes. Hsp70s transiently interact with a wide variety of client proteins, impinging on virtually all stages of a protein’s lifetime (Kampinga and Craig 2010; Kim et al. 2013). They promote protein folding, prevent protein aggregation, foster protein degradation, disassemble protein complexes, and modulate protein–protein interactions. Given their diverse and central functions in cell physiology and protein homeostasis, it is not surprising that Hsp70 chaperone systems have been connected to a number of disease states, including protein misfolding diseases (Broadley and Hartl 2009).

Despite functional diversification among Hsp70s, fundamental sequence and structural features have been conserved. All Hsp70s possess a highly conserved, regulatory N-terminal nucleotide-binding domain (NBD) of approximately 40 kDa and a less well conserved, approximately 25 kDa, C-terminal substrate-binding domain (SBD), which contains the binding site for client proteins (Mayer and Bukau 2005). At its core, Hsp70 chaperone activity is characterized by a cyclic bind and release interaction with client proteins, which is regulated by the ATP binding and hydrolysis activity of the NBD. A conformational change generated upon ATP hydrolysis stabilizes Hsp70s’ interaction with its client protein; exchange of ADP for ATP promotes client release and completes the binding cycle.

Hsp70s do not function alone, but rather as part of complex machinery. The client protein-binding and release cycle requires the activity of co-chaperones: J-proteins and nucleotide release factors. J-proteins stimulate Hsp70s’ ATPase activity, promoting client binding. Nucleotide release factors stimulate release of ADP, and thus facilitating ATP binding and release of client, and as a result promoting initiation of a new Hsp70–client interaction cycle. Those Hsp70s capable of performing the full bind and release cycle with client proteins are termed “canonical.” Despite sequence and structural similarity with canonical Hsp70s, some members of this family have a limited ability to carry out the full binding and release cycle, and are termed “atypical” (Shaner and Morano 2007). Atypical Hsp70s are known to act as co-chaperones for specific canonical Hsp70 partners, acting as nucleotide release factors themselves or as modulators of J-protein co-chaperone activity.

Although Hsp70s are among the most studied genes at the molecular and cellular level, their evolutionary history is not well understood. It is known that Hsp70 gene copy number is dynamic among eukaryotes species, for example, 14 in *Saccharomyces cerevisiae*, 14 in *Caenorhabditis elegans*, 11 in *Drosophila melanogaster*, and 17 in *Homo sapiens* (Daugaard et al. 2007). The most detailed functional and evolutionary data for eukaryotes are available for *S. cerevisiae* (Kampinga and Craig 2010). In this species, the ten canonical members of the Hsp70 family form four subfamilies, which were originally designated based on a combination of cellular location, genetic analysis, and rudimentary sequence comparisons (Boorstein et al. 1994). Two subfamilies are present in the cytosol: four paralogous SSA proteins reside in the nucleus, as well as the cytosol; two paralogous SSB proteins bind cytosolic ribosomes near the polypeptide exit site. Two subfamilies are strictly localized within organelles: A single KAR protein is found in the lumen of the endoplasmic reticulum (ER); three paralogous SSC proteins localize within the mitochondrial matrix. Atypical *S. cerevisiae* Hsp70s form three subfamilies: A single LHS protein functions as a nucleotide release factor for KAR in the lumen of the ER (Steel et al. 2004); two paralogous SSE proteins are nucleotide release factors for both SSA and SSB proteins (Raviol et al. 2006); a single SSZ protein forms a heterodimer with the J-protein co-chaperone of SSB proteins, Zuo1, regulating its ability to stimulate SSB’s ATPase activity (Huang et al. 2005).

Previous studies aimed at understanding the evolutionary diversification of the Hsp70 superfamily predate the genomic revolution (Hughes 1993; Gupta and Singh 1994; Gupta et al. 1997; Germot and Philippe 1999; Bettencourt and Feder 2001; Lin et al. 2001; Nikolaidis and Nei 2004; Brocchieri et al. 2008). Thus, sequence data available for analysis were limited. The recently completed genome sequencing for 53 species of the ancient fungal clade Ascomycota provides a unique opportunity to determine patterns of gene gain and gene loss, as well as mechanisms of gene family evolution (Dujon 2010). Genomic sequence divergence between the distantly related Ascomycota species *S. cerevisiae* and *Schizosaccharomyces pombe* is nearly as great as that between either of these fungi and human, thus providing a deep evolutionary history for examination (Taylor and Berbee 2006). Advantageously, Ascomycota species have a small genome size, relatively complete genome sequence assemblies are available due to low numbers of repetitive sequences in these species, and synteny data are available for many clades.

We found that the Hsp70s present in Ascomycota comprise seven monophyletic subfamilies that corresponded with previous functional categorization in *S. cerevisiae* based on subcellular localization and function. All genomes harbored at least one member from each subfamily, indicating that seven homologs constitute the minimal number of Hsp70s required for proper cell function in Ascomycota. However, despite the uniformity of subfamily composition, dramatic differences with respect to gene copy number dynamics and the evolutionary mechanisms behind duplicate gene retention were uncovered within each subfamily.

## Materials and Methods

### Identification of Hsp70 Orthologs

Genomic data were obtained from the NCBI Genbank, BROAD Institute, *Saccharomyces* Genome Database, Genolevures, DOE Joint Genome Institute, EBI Integr8, Ensembl Genomes, Yeast Gene Order Browser, Genoscope, Sanger Institute, the *Podospora anserina* genome project, the *Saccharomyces* sensu-stricto resequencing efforts (Scannell et al. 2011), and the lab of Cécile Neuvéglise (Neuvéglise C, unpublished data). Contig, scaffold, and open reading frame (ORF) data for each species were examined in local Blast databases (Altschul et al. 1990). To assess the presence or absence of Hsp70 orthologs in a species, BlastP and TBlastN searches were performed using protein sequences of *S. cerevisiae* Hsp70s:Ssa1p (YAL005C), Ssa2p (YLL024C), Ssa3p (YBL075C), Ssa4p (YER103W), Ssb1p (YDL229W), Ssb2p (YNL209W), Kar2p (YJL034W), Ssc1p (YJR045C), Ssc3p/Ecm10p (YEL030W), Ssq1p (YLR369W), Sse1p (YPL106C), Sse2p (YBR169C), Ssz1p (YHR064C), and Lhs1p (YKL073W) as queries. The genomic locations of the best hits obtained using BlastP were cross-validated with the corresponding location of the best hits in raw genomic data obtained using TBlastN. If a high-scoring hit could not be identified within the annotated ORFs, predicted ORFs were annotated manually using ORF finder from highest scoring genomic regions (http://www.ncbi.nlm.nih.gov/gorf/gorf.html, last accessed December 11, 2013). Subcellular localization of the analyzed Hsp70 homologs was predicted using the TargetP v1.1b software (Emanuelsson et al. 2007).

### Sequence Alignments and Phylogenetic Reconstruction

Nucleotide and amino acid sequences of the Hsp70 homologs were used to construct multiple sequence alignments using MAFFT v7 (Katoh and Standley 2013). Alignments generating using the E-INS-i, L-INS-i, and G-INS-i algorithms and the auto option were compared, and the alignment with the largest number of nongap positions was selected for further analyzes. Phylogenetic analyses were carried out using both maximum likelihood (ML) and Bayesian inference (BI) methods. The ML analyzes were performed using RAxML v7.4.2 (Stamatakis 2006) with 100 rapid bootstrap replicates, under the LG model of amino acid substitution with empirical amino acid frequencies and 4 gamma distribution rate categories to estimate rate heterogeneity (LG + G + F) (Le and Gascuel 2008), which was estimated as the best-fitting substitution model by ProtTest v3 (Darriba et al. 2011). The BI analyses were performed using MrBayes v3.2 (Ronquist et al. 2012) under the mixed protein model of amino acid substitution and assuming a gamma distribution of rate variation across sites. The Bayesian analyses were performed with two independent runs, each with four chains, sampling every 100 generations. The analyses were run for 4,000,000 generations and the first 25% of sampled trees were discarded as burn-in before estimation of posterior probabilities for branch support. The calculations were accelerated using the BEAGLE library for Statistical Phylogenetics (Ayres et al. 2012). Validation that the MCMC chains reached stationarity was validated through a combination of PRSF values near 1.0 from the saved generations, standard deviation of the split frequencies below a value of 0.001 from the saved generations, and the two independent runs converged on the same tree topology.

### Sequence Analyses

Sequence identity levels were calculated as the arithmetic mean of all pairwise sequence comparisons in the untrimmed multiple sequence alignment of a given Hsp70 subfamily. Amino acid usage was measured as the number of different amino acids across all nongap sites in the multiple sequence alignment of a given Hsp70 subfamily. Analysis of amino acid sequence divergence of a given Hsp70 subfamily was performed by comparing two groups of Hsp70 sequences: group A (reference group) and group B (analyzed group). In the case of analyses comparing members of the *SSQ1* and *SSC3* subfamilies, the sequences from group A and group B were aligned together. In the cases of analyses comparing members of the SSE, SSZ, and LHS subfamilies, the reference group A constituted of sequences of three canonical Hsp70s subfamilies (SSA, KAR, and SSC). All multiple sequence alignments were manually edited to remove sites containing gaps. Next, sequences from the analyzed group B, were added to the group A alignment using the “–add” option in MAFFT v7 (Katoh and Standley 2013). This option kept the group A alignment unchanged, with the exception of group-wide indels, as the group B sequences were aligned to it. Sequence positions in divergence analyses were standardized relative to analyzed group B sequences from *S. cerevisiae* subfamily homologs. Sites from each group were classified as either invariant, while also allowing for a single amino acid variant within a group, or variable. Sites were classified as conserved were invariant across both groups whereas relaxed sites were invariant in reference group A, but variable in analyzed group B, fixed; sites were variable in reference group A and invariant in analyzed group B, and switched sites were invariant within each group, but contained different amino acids. Sites with gaps or those that were variable in both groups were ignored. Sites from the fixed and switched classes were further subclassified as conservative or radical, based on the BLOSUM62 matrix. If the BLOSUM62 substitution score for the two most prevalent amino acids in group A and group B was strictly larger than zero, the site was classified as conservative. If the score was zero or negative, the site was classified as radical. All analyzes of sequence divergence were performed using scripts written in BioPython (Cock et al. 2009).

### Codon Usage Bias

Codon usage bias was estimated using the effective number of codons (ENC) (Wright 1990), and the Codon Adaptation Index (CAI) (Sharp and Cowe 1991). Both statistics were calculated using the CodonW software (Peden 1999) for orthologs from five closely related *Saccharomyces* sensu-stricto species (*S. cerevisiae*, *S. paradoxus*, *S. mikatae*, *S. kudriavzevii*, and *S. uvarum*). CAI values were calculated using the reference set of optimal codons from *S. cerevisiae*.

### Evolutionary Analyses

Synonymous (d*S*) and nonsynonymous (d*N*) substitution rates for the 14 Hsp70 orthologs from five closely related *Saccharomyces* sensu-stricto species (*S. cerevisiae*, *S. paradoxus*, *S. mikatae*, *S. kudriavzevii*, and *S. uvarum*) were determined using the ML approach as implemented in the PAML package v4.7 (Yang 2007) using the codeml program. A single d*N*/d*S* value was assumed for the entire tree obtained with ML methods and mean d*N* and d*S* rates were obtained by averaging them across all branches of the tree. In all PAML analyses, the codon frequencies were averaged from nucleotide frequencies at the three codon positions (codon frequency model F3x4). To avoid falling into suboptimal likelihood peaks and ensure proper convergence, the analyses were carried out multiple times, using various initial settings of the estimated parameters (d*N*/d*S* ratio and transition to transversion rate kappa, κ). Initial values of 0.1, 0.5, 1, and 5 were used for both parameters in all 16 different combinations and the final likelihood values from all runs were required to be identical. Statistical significance at *P* = 0.05 for differences in codon usage and evolutionary rates was performed using the R package (R Development Core Team 2013), where the distributions of parameter estimates were tested for nonnormality using the Shapiro–Wilk test and the difference between groups was assessed using the Kolmogorov–Smirnov test.

### Species Phylogeny

Species phylogeny was based on Fitzpatrick et al. (2006) for the *Basidiomycota* and *Pezizomycotina*, Rhind et al. (2011) for the *Schizosaccharomyces*, Maguire et al. (2013) for *Candida*, and Gordon et al. (2009) for the *Saccharomycetaceae*. For species closely related to *Yarrowia lipolytica*, a species tree was deduced from the alignment of 912 single copy protein-coding genes (398,959 residues). Individual gene alignments for the 912 orthologs was performed with MUSCLE (Edgar 2004) edited using Gblocks (Castresana 2000) concatenated, and the phylogenetic tree was estimated by ML using PHYML v3.0 (Guindon and Gascuel 2003) assuming a JTT substitution model with gamma-distributed rate variation and a proportion of invariant sites estimated from the data.

### Synteny Analyses

Synteny of Hsp70 orthologs was determined using the Yeast Gene Order Browser (Byrne and Wolfe 2006) for *Saccharomycetaceae* species and the *Candida* Gene Order Browser (Fitzpatrick et al. 2010) for CTG group species. In *Schizosaccharomyces* and *Pezizomycotina*, synteny was assessed by manual annotation and homology comparisons for 20 genes upstream and downstream of each Hsp70 gene. In the *Yarrowia* clade, the genomic location of ten genes upstream and downstream of each Hsp70 gene was compared with *Y. lipolytica.* If synteny was not conserved, then the same analyses were performed with all other *Yarrowia* species.

### Transcription Factor-Binding Sites Analysis

Upstream (5′-intergenomic sequences [5′-IGS]) and downstream (3′-IGS) intergenic sequences of SSB orthologs from post-whole-genome duplication (WGD) species were obtained from the Yeast Gene Order Browser for *S. cerevisiae*, *S. mikatae*, *S. kudriavzevii*, and *S. uvarum*. The 5′-IGS and 3′-IGS of *S. paradoxus* were extracted directly from the genomic data, based on annotation of the surrounding genes. Motifs for 16 transcription factor (TF) binding sites identified previously as present in the promoters of *SSB1* and *SSB2* genes of *S. cerevisiae* were obtained from the study of (MacIsaac et al. 2006). All 5′-IGS of *SSB1* and *SSB2* orthologs from the five post-WGD species were then searched for these motifs using the dna-pattern program from the Regulatory Sequence Analysis Tools (Thomas-Chollier et al. 2008). Motifs with six or fewer bases in length were required to perfectly match the searched sequence, whereas motifs of more than six bases in length were allowed a single base mismatch within the searched sequence.

## Results and Discussion

### Seven Distinct Hsp70 Subfamilies Are Conserved among Ascomycota

**Fig. 1.—:**
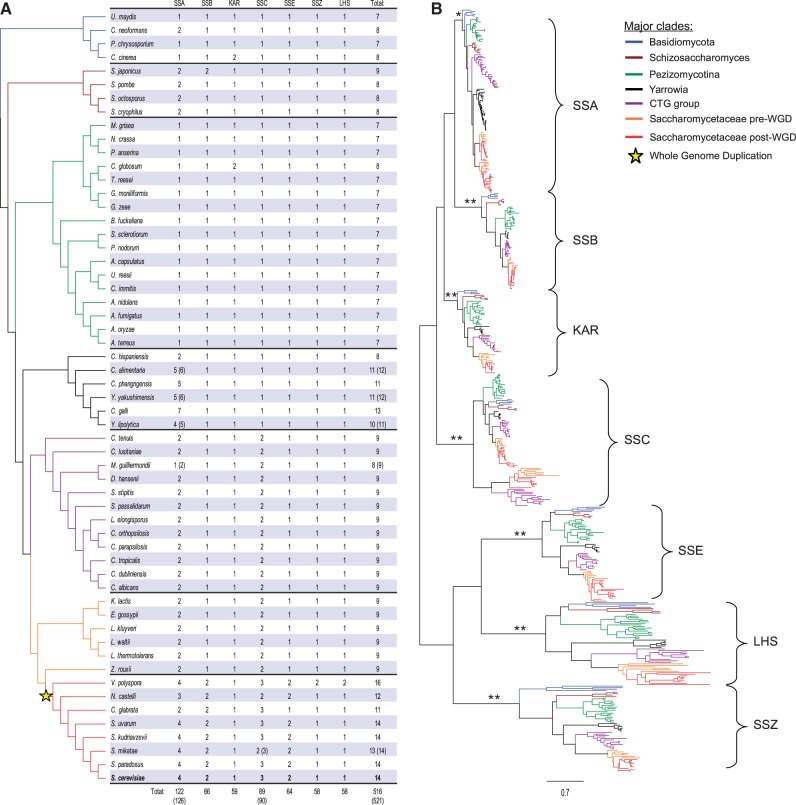
Phylogenetic distribution, copy number dynamics, and protein phylogeny of Hsp70s in fungi. (*A*) Phylogenetic distribution of the Hsp70 orthologs from 7 subfamilies across the surveyed fungal species. Number of orthologs identified for each Hsp70 subfamily is indicated; in parentheses the number of orthologs including putative orthologs (pseudogenes or partial hits due to low quality genomic data (supplementary fig. S1, Supplementary Material online). The SSC column includes orthologs of *SSC3* and *SSQ1* genes. The species tree was based on various sources (see Materials and Methods for details). (*B*) Maximum-likelihood tree of amino acid sequences from all identified Hsp70 orthologs. Scale is in expected amino acid substitutions per site. **Bootstrap ≥ 95, *bootstrap ≥ 70.

As a first step in our evolutionary analysis of Hsp70s in Ascomycota, we sought to identify all Hsp70 genes in 53 Ascomycota genomes. Using the sequences of the 14 *S. cerevisiae* Hsp70s in Blast searches, we identified 491 Hsp70s homologs. Thirty Hsp70 genes were also identified in 4 Basidiomycota genomes for use as an out-group in our protein phylogenetic analysis (fig. 1). We resolved orthologous relationships among Hsp70s through analysis of the reciprocal best Blast scores and the evolutionary position within the phylogeny. In addition, for groups of closely related species, we verified orthology through validation of syntenic relationships among genes. All identified Hsp70 genes were clearly homologous to the well-characterized 14 Hsp70 genes present in *S. cerevisiae* (fig. 1). For clarity throughout this report, we designate identified Hsp70 genes in accordance with the names of their *S. cerevisiae* homologs, with the specific genomic annotations for each identified Hsp70 gene provided in supplementary figure S1, Supplementary Material online.

**Fig. 2.—:**
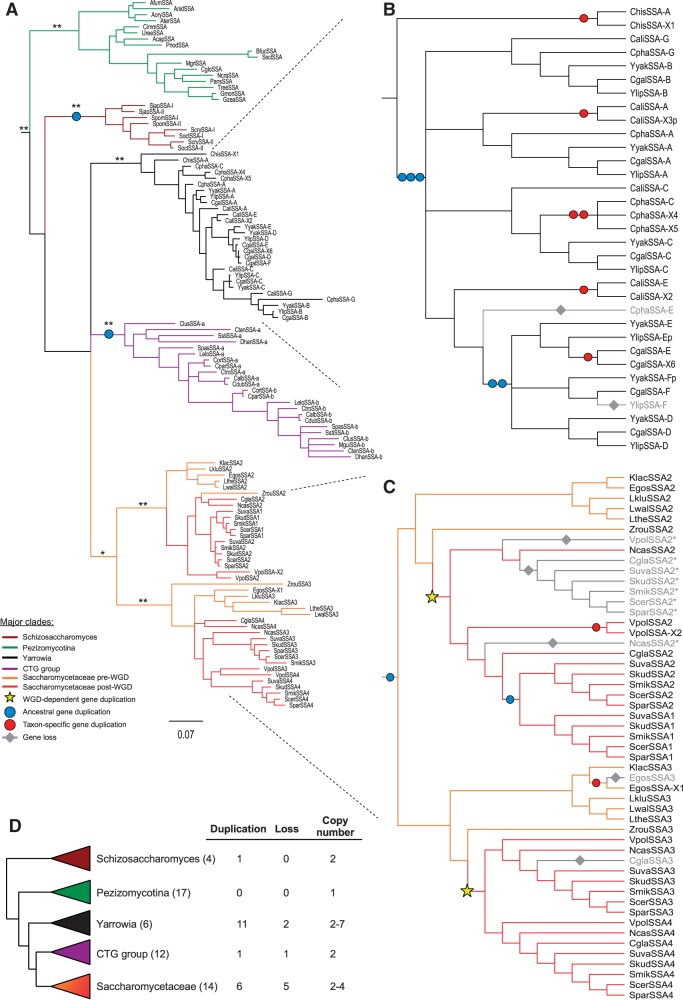
Protein phylogeny and copy number dynamics of the SSA subfamily. (*A*) Bayesian tree of amino acid sequences from the SSA subfamily. Scale is in expected amino acid substitutions per site. **Posterior probability ≥ 0.95, *posterior probability ≥ 0.7. (*B*) Cladogram showing the hypothetical scenario of SSA evolution in the *Yarrowia* clade. Gene copies named A–G exhibit synteny across at least two species, gene copies named X1–X6 are taxon specific and gene copies named “p” are putative pseudogenes. (*C*) Cladogram showing the hypothetical scenario of SSA evolution in the CTG and *Saccharomycetaceae* clades, gene copies named X1 and X2 are taxon specific. (*D*) Copy number dynamics in the SSA subfamily. Number of species for each clade indicated in parenthesis.

Invariably, we found that each surveyed genome harbored homologs of all seven Hsp70 subfamilies that correspond to those recognized in *S. cerevisiae*, that is both the four canonical subfamilies (SSA, SSB, KAR, and SSC) and the three atypical subfamilies (SSE, SSZ, and LHS). Genomes from 16 of the 53 analyzed Ascomycota contained only seven Hsp70 genes, in every case one from each subfamily. For the 37 species having more than seven Hsp70s, gene copy number and evolutionary dynamics varied significantly among Hsp70 subfamilies. As discussed later, some subfamilies have a stable number of paralogous copies, particularly when they resulted from genome-scale duplication events. Other subfamilies have highly variable gene copy number, resulting from independent taxon-specific gene duplications. Regardless of their evolutionary history, each subfamily was recovered as a monophyletic group with strong branch support on the phylogenetic tree, regardless of whether nucleotide or amino acid sequence data sets were examined (fig. 1). Phylogenetic analyses also indicated that within each subfamily, gene trees generally recapitulated the hypothesized species trees (figs. 1 and 2; supplementary figs. S2–S7, Supplementary Material online).

The invariant presence of homologs of the seven subfamilies suggests that they comprised the ancestral combination present in the common ancestor of Ascomycota, and that the seven *S. cerevisiae* subfamilies have been experimentally demonstrated to comprise functionally distinct groups based on both subcellular localization and phenotypic effects of mutations (Kampinga and Craig 2010) supports this idea. This conclusion is also consistent with the previously postulated evolutionary basis for functional diversification of Hsp70 gene family (Boorstein et al. 1994; Daugaard et al. 2007). Thus, the seven Hsp70 subfamilies most likely constitute units of function and evolution conserved across surveyed fungal genomes. To more thoroughly examine evolutionary patterns and modes of sequence evolution within each of these Hsp70 subfamilies, we analyzed them individually by generating separate sequence alignments for each. This approach led to more evolutionarily informative sites for each subfamily, and thus facilitated the more detailed studies described in the following sections.

### Canonical SSA Hsp70s: Dynamic Copy Number Evolution

Among the 53 Ascomycota species surveyed, we identified 117 homologs of SSA with two distinct patterns of gene copy number evolution (figs. 1 and 2*A*). The 17 species of Pezizomycotina all harbored a single SSA ortholog, whereas the 36 species belonging to *Schizosaccharomyces*, *Yarrowia*, CTG group, and *Saccharomycetaceae* had more than one SSA gene. The copy number varied in a taxon-specific manner, with individual genomes harboring between two and seven SSA genes (figs. 1 and 2*A*). The pattern of SSA copy number variation is consistent with the occurrence of several gene duplication-plus-retention events (fig. 2). In some cases, a rather straightforward scenario can be envisioned. For example, two independent ancestral gene duplications, each of which took place in a common ancestor of either the CTG group or *Schizosaccharomyces*, is sufficient to explain the pair of SSA copies present in species belonging to these clades. While a single gene loss that took place in a species specific lineage within the CTG-group, could explain the presence of a single SSA copy in *Meyerozyma guilliermondi*. However, as discussed later, SSA evolution in the *Yarrowia* and Saccharomycetaceae clades exhibited very complex SSA gene dynamics.

Copy number in the *Yarrowia* clade ranges between two and seven among the six species analyzed (fig. 2). The precise branching order among paralogs was not fully resolved, likely because the number of informative amino acid sites was relatively low (supplementary fig. S8, Supplementary Material online). Moreover, a strict interpretation of copy number dynamics based on the Bayesian gene tree resulted in a large number of gene retention and loss events. Therefore, we considered alternative scenarios. Using synteny data to determine the orthologous relationships among the SSA genes within this clade, we identified seven loci (marked as SSA-A to SSA-G, fig. 2*B*) conserved across at least two *Yarrowia* species. These loci allowed us to determine orthologous relationships among 25 of the 31 SSA genes. For the remaining 6 genes (marked as SSA-X1 to SSA-X6, fig. 2*B*), we were unable to identify syntenic conservation; thus their orthologous relationships remained uncertain. To reconstruct an evolutionary scenario that could explain the observed gene copy number dynamics, we placed 25 SSA genes for which orthologous relationships were determined onto the species tree. Next, we added the remaining 6 SSA genes, for which orthologous relationships were not resolved, in a way that minimized the number of necessary evolutionary events (i.e., gene duplication and gene loss) (fig. 2*B*). The results indicated that SSA copy number dynamics in *Yarrowia* clade could be explained by five ancestral duplications in combination with six lineage-specific gene duplications and two losses—A total of 13 events (fig. 2*B* and *D*). Interestingly, one of the ancestral duplications, which took place before the divergence of *Y. lipolytica*, *Y. yakushimensis*, and *C. galli*, generated a subtelomeric copy that was further propagated by either a reciprocal or nonreciprocal translocation to another subtelomere (SSA-D and SSA-F). Additionally in *C. galli*, a third subtelomeric copy (CgalSSA-X6) was identified, corresponding to a species-specific duplication. Based on this analysis, we conclude that lineage-specific gains and losses were exceptionally common for SSA homologs within the *Yarrowia* clade.

To explain even more complex copy number dynamics exhibited by species in the *Saccharomycetaceae* clade, we combined phylogenetic and synteny data with published genomic analyses (Gordon et al. 2009). According to our hypothetical scenario, a gene duplication that took place in the ancestor of *Saccharomycetaceae* accounted for formation of the gene pair orthologous to *SSA2* and *SSA3* of *S. cerevisiae*. Next, both the *SSA2* and the *SSA3* genes were duplicated in the WGD event (Wolfe and Shields 1997) ancestral to the *S. cerevisiae* lineage (fig. 2*C*). The post-WGD fate of *SSA3* was straightforward; the SSA3/SSA4 pair of ohnologs was retained in most post-WGD species, with only *C. glabrata* experiencing a loss of *SSA3*. The evolutionary history of *SSA2* genes in post-WGD species is complex. Both phylogenetic and synteny data are consistent with the occurrence of at least three independent loss events (fig. 2*C*), resulting in only one *SSA2* gene copy being maintained in each species. However, subsequently two independent duplications of *SSA2* gene took place. One was in the ancestor of the *Saccharomyces* sensu-stricto clade, which resulted in formation of the *SSA2*/*SSA1* paralogous gene pair currently present in *S. cerevisiae* and closely related species. An independent, species specific, gene duplication is sufficient to explain the presence of two *SSA2* copies in *Vanderwaltozyma polyspora* (called *SSA2* and *SSA-*X2 in fig. 2*C*). Altogether, based on these analyses we concluded that SSA subfamily experienced highly dynamic copy number evolution driven by genomic and/or gene-specific gain and loss events.

### Canonical SSA Hsp70s: Low Sequence Divergence

**Fig. 3.—:**
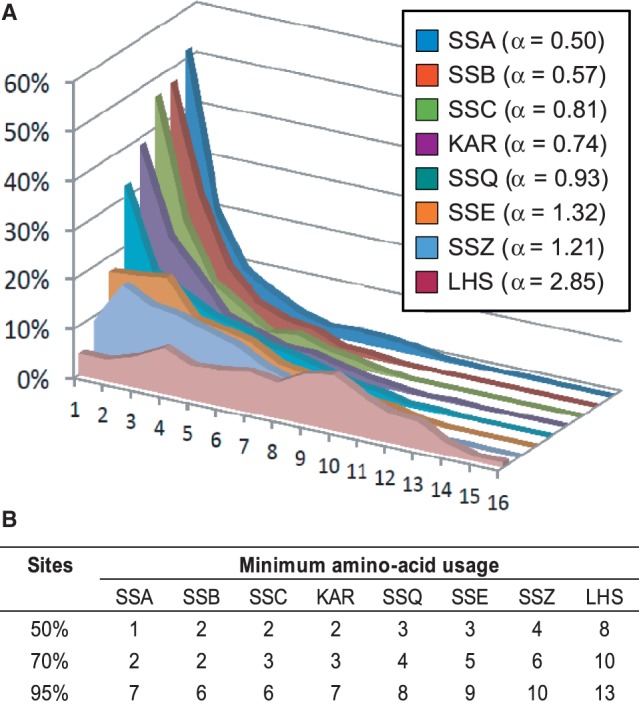
Amino acid usage among Hsp70 subfamilies. (*A*) Distribution of sites with indicated amino acid usage, defined as the number of different amino acids observed per site in a multiple sequence alignment. (Inlet) Values of the shape parameter α of the gamma distribution fitted to the amino acid usage data for each Hsp70 subfamily, as indicated. (*B*) Maximum amino acid usage covering the indicated proportion of sites in each Hsp70 subfamily.

In contrast to the high copy number dynamics, sequence evolution of SSA genes was very slow, as illustrated by the short branches of the protein tree (figs. 1 and 2*A*) and the high level of mean pairwise amino acid sequence identity (∼82%, table 1). To further characterize the mode of sequence evolution, we analyzed the distribution of amino acid usage (*u*), defined as the number of different amino acids observed per site in the sequence alignment (Breen et al. 2012). As the amino acid usage can be used as a proxy for the substitution rate (Breen et al. 2012), we fit a gamma distribution to the data (fig. 3*A*). The gamma distribution has the shape parameter α, which specifies the range of rate variation among sites. An α value lower than 1 indicates an L-shaped distribution, where most sites are invariable. Conversely, an α greater than 1 indicates a bell-shaped distribution, with the majority of sites having higher *u* values.

**Table 1:** Global Analysis of Hsp70 Subfamilies in Ascomycota

Subfamily	Sequence Statistics			Cellular Localization
	No. of Orthologs^a^	Length^b^	Identity^c^	
Canonical				
SSA	117	645 ± 5	81.68 ± 4.81	Cytosol
SSB	61	613 ± 1	80.45 ± 8.80	Cytosol
KAR	53	674 ± 9	72.46 ± 6.99	ER
SSC	53	658 ± 13	74.51 ± 8.08	Mitochondria
SSQ	26	648 ± 8	59.63 ± 10.64	Mitochondria
Atypical				
SSE	60	703 ± 18	55.62 ± 13.13	Cytosol
SSZ	54	550 ± 15	49.44 ± 13.40	Cytosol
LHS	52	947 ± 61	26.42 ± 12.76	ER

^a^Number of identified orthologs, excluding putative orthologs and sequences with unique internal deletions. ^b^Mean sequence length ± SD.

^c^Mean pairwise amino acid sequence identity ± SD.

For SSA homologs, the distribution of amino acid usage was markedly L shaped (α = 0.50, fig. 3*A*), indicating that for most sites the *u* value was very low (supplementary fig. S9, Supplementary Material online). For example, for 50% of sites *u* was equal to 1, indicating that those sites were invariant; for 70% of sites *u* was lower or equal to 2 showing that, at most, one alternative amino acid was present in the sequence alignment (fig. 3*B*). These observations are consistent with a high level of constraint of the sequence variation, caused by strong purifying selection.

To test whether the SSA sequences were subjected to the same evolutionary selection pressures over a short timescale, we analyzed synonymous (d*S*) and nonsynonymous (d*N*) substitution rates for a set of five species closely related to *S. cerevisiae* (table 2) for which detailed genomic analysis are available (Scannell et al. 2011). As d*S* values were not saturated for any Hsp70 sequence (d*S *≪ 1) in this data set, we were able to calculate d*N*/d*S* ratios for all members of all Hsp70s subfamilies (table 2). Each of the five analyzed genomes harbors four paralogous copies of SSA genes, with *SSA3* and *SSA4* originating from the WGD and *SSA1* and *SSA2* from the subsequent lineage-specific gene duplication event (fig. 2). The two pairs of SSA paralogs share very low d*N*/d*S* values (table 2). In fact their d*N*/d*S* values were from 4- to 10-fold lower than those estimated for average protein coding gene harbored by the five analyzed species (Scannell et al. 2011). Yet, the two pairs of SSA paralogs differ markedly in their sequence divergence, with *SSA1* and *SSA2* sequences sharing a high percentage of invariant codons (56% and 67%, respectively) and a highly biased codon usage (table 2). In contrast, the percentage of invariant codons was approximately 2-fold lower for the *SSA3* and *SSA4* pair and they exhibited much less biased codon usage. The higher sequence divergence of *SSA3/SSA4* pair could have resulted from longer evolutionary history due to their earlier origin (fig. 2*C*), whereas their higher codon bias could be explained by their relatively low expression levels observed under optimal growth conditions (table 2). However, upon stress conditions or during transition to the stationary phase (the diauxic shift) all four SSA paralogs are expressed at comparable level (Werner-Washburne et al. 1989), indicating that more complex mechanisms could be responsible for the observed differences in codon bias. Yet, overall, our data suggest that at both long- and short-term evolutionary timescales, members of SSA subfamily evolved very slowly.

**Table 2:** Codon Usage and Evolutionary Rates of the Hsp70 Genes in *Saccharomyces cerevisiae*, *S. paradoxus*, *S. mikatae*, *S. kudriavzevii*, and *S. uvarum*

Hsp70	Codon Usage					Protein Abundance^d^	Evolutionary Rates		
	Total^a^	Invariant^b^	% Invariant	CAI^c^	ENC		d*N*	d*S*	d*N*/d*S*
SSA1	642	361	56.2	0.65 ± 0.06	29.85 ± 1.34	8,178	0.003 ± 0.002	0.259 ± 0.169	0.013
SSA2	639	426	66.7	0.78 ± 0.02	27.40 ± 0.43	7,986	0.002 ± 0.001	0.234 ± 0.110	0.010
SSA3	647	188	29.1	0.19 ± 0.01	47.29 ± 3.20	290	0.007 ± 0.003	0.292 ± 0.127	0.025
SSA4	642	175	27.3	0.21 ± 0.03	43.09 ± 1.57	475	0.006 ± 0.004	0.331 ± 0.220	0.019
SSB1	613	420	68.5	0.80 ± 0.02	27.57 ± 0.71	2,320	0.002 ± 0.001	0.186 ± 0.122	0.010
SSB2	613	405	66.1	0.77 ± 0.02	27.83 ± 0.40	2,791	0.002 ± 0.001	0.176 ± 0.083	0.012
KAR2	682	300	44.0	0.45 ± 0.04	35.81 ± 0.74	523	0.004 ± 0.003	0.238 ± 0.145	0.018
SSC1	651	285	43.8	0.51 ± 0.02	35.05 ± 1.36	709	0.004 ± 0.003	0.257 ± 0.186	0.016
SSQ1	654	185	28.3	0.14 ± 0.01	49.93 ± 2.62	26.4	0.014 ± 0.006	0.307 ± 0.123	0.045
SSC3 (d)^e^	643	181	28.1	0.20 ± 0.01	47.50 ± 0.92	86	0.027 ± 0.011	0.372 ± 0.151	0.073
SSC3 (c)^f^	630	281	44.6	0.52 ± 0.04	34.24 ± 0.31	n/a	n/a	n/a	n/a
SSE1	693	296	42.7	0.55 ± 0.05	34.04 ± 1.70	1,959	0.007 ± 0.004	0.289 ± 0.178	0.025
SSE2	693	180	26.0	0.20 ± 0.01	48.91 ± 1.62	155	0.017 ± 0.009	0.319 ± 0.170	0.053
SSZ1	538	186	34.6	0.44 ± 0.05	36.02 ± 2.09	1,040	0.011 ± 0.007	0.274 ± 0.194	0.038
LHS1	881	191	21.7	0.15 ± 0.01	50.69 ± 2.08	20.3	0.038 ± 0.022	0.302 ± 0.179	0.125

Note.—n/a, not applicable.

^a^Total number of aligned codons.

^b^Number of identical codons in the analyzed species.

^c^CAI shown as mean ± SD, for statistical significance see supplementary figure S10, Supplementary Material online.

^d^Protein abundance in parts-per-million (Wang et al. 2012).

^e^SSC3 (d)—divergent *SSC3* orthologs from *S. cerevisiae*, *S. paradoxus*, *S. kudriavzevii*, and *S. uvarum*, that form a separate clade on the phylogenetic tree of the SSC subfamily (supplementary fig. S4, Supplementary Material online).

^f^SSC3 (c)—gene-converted *SSC3* orthologs from *Candida glabrata* and *Vanderwaltozyma polyspora*, that cluster with SSC1 orthologs from their respective species on the phylogenetic tree of the SSC subfamily (supplementary fig. S4, Supplementary Material online).

We also examined whether concerted evolution, driven by gene conversion events, could explain the high sequence identity observed among SSA subfamily members. In cases of concerted evolution paralogous sequences from within a species form sister branches on a gene tree, indicating that they are more similar to each other than to their orthologs from other species (Dover 1982). Such signs of concerted evolution were detected in only two cases for SSA subfamily members (three SSA paralogs in *Y. yakushimensis* and three paralogs in *C. galli*), both of which belong to the *Yarrowia* clade (supplementary fig. S8, Supplementary Material online). Interestingly, all but two of these genes (*YyakSSA-E*, *YgalSSA-E)* are located in the subtelomeres. In other species, including five closely related to *S. cerevisiae*, the branching order of the SSA subfamily gene tree recapitulated the topology of the species tree (figs. 1 and 2), indicating that they indeed evolved according to a divergent mode of sequence evolution under purifying selection.

Overall, the pattern of SSA evolution is consistent with the birth-and-death evolutionary scenario for gene family evolution (Nei and Rooney 2005), wherein gene duplicates are functionally equivalent, so that retention and loss of paralogs is a stochastic process. We hypothesized that harboring multiple paralogs with partially overlapping functions may provide increased robustness of this major cytosolic chaperone system both under physiological and stress conditions. Consistent with this hypothesis, functional data from *S. cerevisiae* indicate that members of SSA subfamily form a dynamic functional network wherein depletion of one paralog leads to compensatory induction of another (Werner-Washburne et al. 1987). Yet, despite a high degree of sequence identity relatively to typical protein coding genes, members of the SSA subfamily also have distinct functional properties. In *S. cerevisiae* SSA paralogs differ in patterns of expression, with *SSA1/SSA2* being expressed constitutively while *SSA3/SSA4* induced by stress (Werner-Washburne et al. 1987). Moreover, *SSA1/SSA2* copies differ in their ability to assist in protein translocation from the cytosol into the ER and/or vacuole (Deshaies et al. 1988; McClellan et al. 1998; Brown et al. 2000), as well as in prion propagation (Sharma and Masison 2011). Similarly, data for *Y. lipolytica* indicate functional diversification among four SSA paralogs within this species (Sharma et al. 2009).

Dynamic copy number evolution of the SSA subfamily is not only restricted to fungi, but also has been observed in *Drosophila* and mammals (Bettencourt and Feder 2001). In contrast to fungi, the SSA genes from *Drosophila* evolved in a concerted manner via gene conversion (Bettencourt and Feder 2002). The authors hypothesized that multiplication of SSA genes in *Drosophila* species may be adaptive because the resulting increased expression provides resistance to a variety of types and severity of stresses. They also note, however, that other species of *Drosophila* have achieved high Hsp70 expression and stress resistance with a limited number of SSA genes. Thus, there is no simple functional explanation for the multiple copies of SSA harbored by *Drosophila* genomes. In humans, six paralogous members of SSA subfamily exhibit complex pattern of evolution and functional diversification with approximately half of them being stress inducible and the other half expressed constitutively (Daugaard et al. 2007). Two of the stress inducible genes (*HSPA1A/B*) are evolving by concerted evolution, driven by high GC content and biased gene conversion (Kudla et al. 2004). In summary, the slow rate of sequence evolution on a global scale that is characteristic for SSA homologs could be explained by purifying selection due to strong functional constraints imposed by the multiple roles they perform in the cytosolic/nuclear compartment of all eukaryotic cells. However, understanding the functional variation among SSA lineages also requires comparisons of gene-specific evolutionary patterns among closely related species. Further studies combining evolutionary analysis with experimental approaches will likely be key in answering the question of whether SSA paralogs have diversified functionally or whether their different expression patterns evolved to allow for differences in regulation of proteins having the same biochemical function (Harms and Thornton 2013).

### Canonical SSB Hsp70s: Stable Copy Number with Concerted Evolution

In contrast to SSAs, there were few evolutionary changes in copy number in the SSB subfamily. A total of 62 orthologous sequences were identified in the 53 surveyed Ascomycota species (fig. 1 and supplementary fig. S2, Supplementary Material online). The presence of *SSB1/SSB2* paralogs in the 8 species of the *Saccharomycetaceae* lineage is consistent with a single gene duplication event that coincided with the WGD. Two copies of SSB were also found in the recently sequenced genome of *Schizosaccharomyces japonicus* (supplementary fig. S2, Supplementary Material online), whereas a single copy was present in the other 44 genomes surveyed.

**Fig. 4.—:**
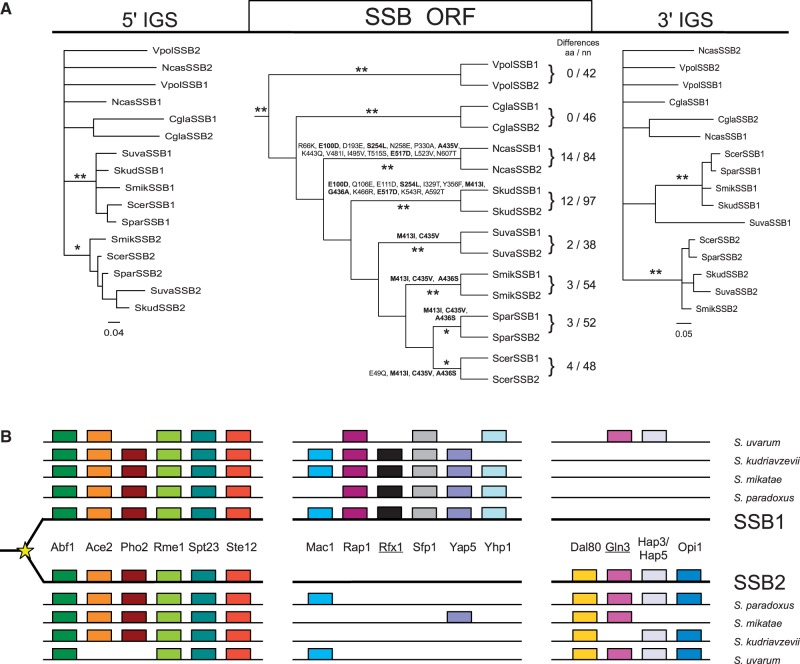
Sequence evolution and regulatory divergence of SSB subfamily. (*A*) Bayesian trees of nucleotide sequences of SSB orthologs from post-WGD *Saccharomycetaceae* species: (left) the 5'-IGS, (middle) open reading frames (SSB ORF), and (right) the 3'-IGS. The SSB ORF tree was rooted using the *SSB1* ortholog from *Candida albicans* and is shown as cladogram for clarity (for the corresponding phylogram, see supplementary fig. S2*B*, Supplementary Material online). Scale is in expected nucleotide substitutions per site. **Posterior probability ≥ 0.95, *posterior probability ≥ 0.7. Differences aa/nn indicates number of amino acid (aa) and nucleotide (nn) differences between paralogous *SSB1/SSB2* sequences from a given species. Residues listed along branches indicate amino acid substitutions between each *SSB1* and *SSB2* pair. Substitutions present in more than one species are marked in bold. (*B*) Schematic representation of predicted TF-binding sites present in the 5'-IGSs of *SSB1* and *SSB2* orthologs from the indicated post-WGD species. Underlined TF-binding sites were not identified in the pre-WGD species. The diagram is not drawn to scale. For clarity, specific locations of individual TF binding sites are not indicated.

Similar to SSAs, SSB sequences evolved slowly, as illustrated by a high pair wise sequence identity (∼80%) (table 1) and an L-shaped distribution of amino acid usage, with α markedly lower than 1 (fig. 3). Also indicative of a divergent mode of sequence evolution controlled by purifying selection, the tree topology for singleton SSB genes was consistent with the species tree (fig. 1; supplementary fig. S2, Supplementary Material online). However, the amino acid phylogeny of paralogous *SSB1/SSB2* sequences from post-WGD *Saccharomycetaceae* species show a complex branching pattern not consistent with a divergent mode of sequence evolution (supplementary fig. S2*A*, Supplementary Material online). As this could be due to weak phylogenetic signal resulting from high level of sequence conservation, we turned to nucleotide-based phylogeny (fig. 4 and supplementary S2*B*, Supplementary Material online). The tree topology for paralogous *SSB1/SSB2* sequences exhibited a branching pattern characteristic of concerted evolution. The high sequence similarity between duplicate SSB paralogs via concerted evolution is consistent with the gene dosage hypothesis (Sugino and Innan 2006), wherein selection acts to maintain duplicate genes for increased protein concentrations. SSBs, which assist folding of polypeptide chains emerging from the ribosome, fit this criterion because their abundance is so high that it equals or surpasses that of ribosomes in the cell (Pfund et al. 1998).

However, SSB evolution was clearly more complicated than can be accommodated by the simple form of dosage hypothesis. Interestingly, the nucleotide sequences just outside of the *SSB1/SSB2* coding sequences are highly variable within species and the phylogeny of their 5'- and 3'-IGS exhibit a branching pattern characteristic of divergent evolution (fig. 4*A*). To examine whether SSB paralogs exhibit evidence of potential divergent transcriptional profiles, we searched for putative TF-binding sites in 5'-IGS regions of *SSB1/SSB2* genes from five species closely related to *S. cerevisiae.* We focused on *S. cerevisiae* TFs for which functional data are available (MacIsaac et al. 2006). Among 16 identified TF-binding sites (supplementary fig. S11, Supplementary Material online), we observed two distinct patterns of presence/absence within the 5'-IGS sequences of *SSB1/SSB2* paralogs. Six of these TF-binding sites are shared between the 5'-IGS regions of both *SSB1/SSB2* copies (fig. 4*B*). The remaining ten exhibit a divergent pattern with six TF-binding sites being present predominantly upstream of *SSB1*, while the other four are present predominantly upstream of *SSB2*. Moreover, while 14 of the 16 TF binding sites are also present among the 5'-IGS regions of singleton SSB genes from species ancestral to the WGD event (Kominek J, unpublished data), two are present only in post-WGD species. Namely, the Rfx1 binding site is present in 5'-IGSs of *SSB1*, while the Gln3-binding site is present in 5'-IGS of *SSB2* in 4 out of the 5 species, suggesting that further functional diversification may have taken place after emergence of SSB duplicates.

The earlier discussion focuses on the possible functional identity of the *SSB1/SSB2* paralogs. However, a degree of functional specialization may exist despite their high level of sequence identity. A few of the amino acid substitutions exhibit repeated instances of parallel evolution among paralogous copies of SSBs (fig. 4*A*), implying, as suggested previously (Takuno and Innan 2009), that these substitutions could have functional importance and were thus driven by selection. However, we note that two species (*V. polyspora* and *C. glabrata*) harbor copies of the *SSB1/SSB2* gene pair having identical amino acid sequences. One possible explanation is that the identity in these species is due to recent gene conversion events. On the other hand, as the species harboring identical copies of *SSB1/SSB2* genes branch at the base of the clade harboring SSB duplicates, it is also possible that functionally important sequence divergence of *SSB1/SSB2* copies evolved more recently.

Overall, SSB subfamily evolution is a remarkable example of independent gene conversion, where the physical boundaries of each event correspond precisely with the coding region of each duplicate pair. Noncoding sequences adjacent to both sides of the coding region exhibit clear patterns of divergent evolution, but closer inspection of promoter regions shows evidence of potential subfunctionalization for regulatory elements following gene duplication. Finally, despite concerted evolution within coding sequences, multiple instances of parallel amino acid substitutions indicate some degree of functional divergence between gene duplicates. Further functional studies are needed to fully explain the biological importance of the complex evolutionary patterns observed for *SSB1/SSB2* gene duplicates

### Canonical KAR Hsp70s: Stable Gene Copy Number and Divergent Sequence Evolution

In the 53 Ascomycota genomes surveyed, we identified only 54 copies of KAR orthologs, indicating stable gene copy number in the KAR subfamily (fig. 1; supplementary fig. S3, Supplementary Material online). The single, species specific, KAR duplicate was identified in *Chaetomium globosum* (fig. 1). The rate of sequence evolution of KAR is low, as indicated by high pairwise protein sequence identity (∼72%) among KAR orthologs (table 1), the L-shaped amino acid usage distribution with α = 0.74 (fig. 3), and d*N*/d*S* value (0.018) (table 2). In summary, KAR is the most stable subfamily among the canonical Hsp70s in Ascomycota.

This stability of KAR is consistent with observations in other lineages, as single orthologs have been identified in a wide range of eukaryotes, including human (Daugaard et al. 2007), *Xenopus* (Miskovic et al. 1997), several fish species (He et al. 2013), *Drosophila* (Nikolaidis and Nei 2004), silkworm (Xi and Ma 2013), copepod (Xuereb et al. 2012), mollusks (Clark et al. 2008), shrimp (Luan et al. 2009), Giardia (Gupta and Singh 1994), *Trypanosoma cruzi* and *Leishmania major* (Louw et al. 2010). Taxon-specific duplications of KAR genes have been identified in *Caenorhabditis* (Nikolaidis and Nei 2004) and in *Trypanosoma brucei* (Louw et al. 2010). However, in flowering plants, an expansion of the KAR subfamily has been observed, with copy number ranging between two and five among the six species analyzed (Denecke et al. 1991; Marocco et al. 1991; Cascardo et al. 2000; Lin et al. 2001; Jung et al. 2013). Thus, even the most stable subfamily of canonical Hsp70s occasionally exhibits copy number variation in a lineage specific manner.

### Canonical SSC Hsp70s: Complex Evolutionary History Involving Subfunctionalization

Our survey of 53 Ascomycota genomes revealed 86 members of the SSC subfamily. Although 27 species harbor a single copy of this mitochondrial Hsp70, those belonging to the CTG and *Saccharomycetaceae* clades bear either 2 or 3 (fig. 1; supplementary fig. S4, Supplementary Material online). Consistent with our previous, more limited, analysis (Schilke et al. 2006), two independent gene duplication events are sufficient to explain this phylogenetic distribution. First, a duplication in a common ancestor of the CTG and *Saccharomycetaceae* clades could explain the presence of the two paralogous SSC genes in members of the CTG clade and those *Saccharomycetaceae* species that did not experience the WGD event. A subsequent duplication is consistent with the presence of three paralogous SSC genes in *Saccharomycetaceae* post-WGD species. More specifically, the ancestral duplication of *SSC1* gave rise to *SSQ1*, and the subsequent WGD gave rise to *SSC3* (known also as *ECM10*). The phylogeny is consistent with this pattern in that *SSQ1* is derived within *SSC1* and *SSC3* forms a sister taxon with *SSC1* (supplementary fig. S4, Supplementary Material online).

Singleton sequences of the SSC subfamily evolved slowly, as is typical for canonical Hsp70s from Ascomycota (tables 1 and 2; fig. 3). However, sequences orthologous to *SSQ1* evolved quite rapidly, relative to other canonical Hsp70s, as indicated by: 1) longer branches on the gene tree (fig. 1; supplementary fig. S4, Supplementary Material online), 2) lower pair-wise amino acid identity (∼60%; table 1), 3) higher d*N*/d*S* values (0.045; table 2), and 4) among all canonical Hsp70s, the highest value of α (0.93; fig. 3) for amino acid usage. The increased rate of substitution for the derived SSQ1 suggests less purifying selection in this subclade (fig. 3).

Two causative factors are consistent with the higher rate of *SSQ1* sequence evolution: 1) low levels of expression (table 2) and 2) highly specialized function. Given the established negative correlation between the rate of sequence evolution and protein expression level (Drummond et al. 2005), the low level of expression of *S. cerevisiae SSQ1* compared with *SSC1* (table 2) provides one possible explanation. In addition, detailed functional studies of *SSQ1* from *S. cerevisiae* revealed that it underwent subfunctionalization, wherein it became specialized in the maturation of iron–sulfur cluster proteins, which had been carried out by the ancestral *SSC1* gene (Schilke et al. 2006). During its subfunctionalization, *SSQ1* lost functions normally carried out by *SSC1*, such as protein import across the inner mitochondrial membrane, protein folding, and refolding of damaged proteins. These diverse functions require *SSC1* to interact with an array of client proteins and several J-protein co-chaperones (Craig and Marszalek 2011). In contrast, biochemical experiments revealed that *SSQ1* interacts with a single client, a scaffold protein on which iron–sulfur clusters are formed, and that this specific interaction requires cooperation with only a single specialized J-protein Jac1(Craig and Marszalek 2011). As *SSQ1* lost the ability to interact with other clients or J-protein co-chaperones, we hypothesize that fewer functional constraints acting upon *SSQ1* resulted in relaxed purifying selection and increased rate of sequence evolution observed.

**Fig. 5.—:**
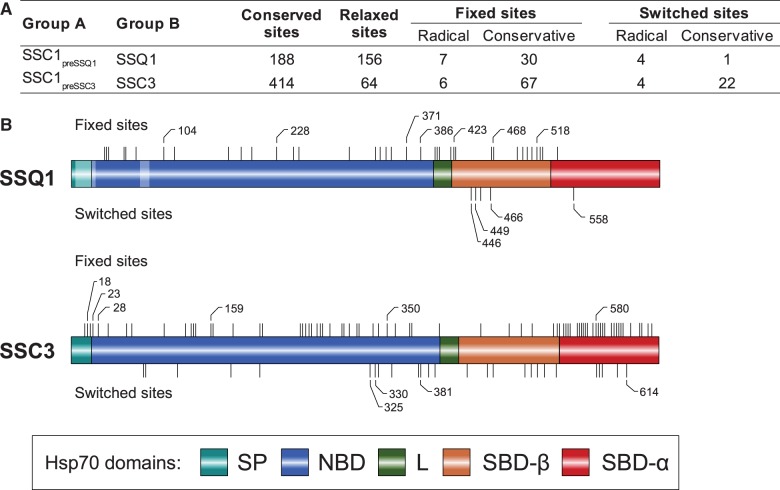
Amino acid sequence divergence of *SSQ1* and *SSC3* orthologs. (*A*) Summary of sequence divergence analysis. Group A: SSC1_preSSQ1_—27 sequences of *SSC1* orthologs from species predating the emergence of *SSQ1*; SSC1_preSSC3_—6 sequences of *SSC1* orthologs from species predating the emergence of *SSC3* (supplementary fig. S4, Supplementary Material online, for details). Group B: 26 sequences of *SSQ1* orthologs and 4 sequences of *SSC3* orthologs. Conserved sites—number of sites with residues identical in group A and B. Relaxed sites—number of sites that are invariant in A but variable in B. Fixed sites—number of sites that are variable in A but invariant in B. Switched sites—number of sites that are invariant within group A and group B but occupied by different amino acids in each group. Radical—number of sites where substitution from the most prevalent residue in group A to the most prevalent residue in group B had the BLOSUM62 score equal to or less than zero. Conservative—number of sites where substitution from the most prevalent residue in group A to the most prevalent residue in group B had the BLOSUM62 score strictly greater than zero. (*B*) Approximate localization of the radical fixed and radical switched sites within sequences orthologous to *SSQ1* and *SSC3* as indicated. Fixed sites represented by bars above the sequence and switched sites marked by bars below sequence with radical sites marked by their sequence position. Pale fragments indicate patches of at least 9 consecutive gaps in the alignment of sequences from groups A and B, and thus not subjected to the analysis. SP, signal peptide; L, Linker; SBD-α, substrate-binding domain alpha-helical lid.

To find signatures consistent with functional diversification of *SSQ1*, we compared *SSQ1* with *SSC1* from species that pre-dated the gene duplication (fig. 5), specifically searching for sites that were variable in *SSC1* but invariant in *SSQ1* (Gu 2001, 1999). We termed such sites “fixed” (called Type I in Gu [1999]), which are interpreted to be potentially important for the specialized function of *SSQ1*. We also searched for switched sites (called Type II in Gu [1999]) that were conserved within each *SSC1* and *SSQ1* sequences, but occupied by different amino acids in each. Type II sites are often interpreted as associated with functional changes in each paralog. The fixed and switched sites were further classified as radical and conservative, based on BLOSUM62 scores. We classified the site as radical when the substitution from the most prevalent residue in the *SSC1* sequences to the most prevalent residue in the *SSQ1* sequences had the BLOSUM62 score equal to or less than zero and conservative when the BLOSUM62 score was strictly greater than zero.

*SSQ1* contains 30 fixed conservative sites that are spatially distributed evenly across the linear amino acid sequence (fig. 5). The spatial distribution of fixed radical sites was very different. Five of seven were within or in close proximity to the client protein-binding cleft localized in the beta sheet region of the SBD domain (SBD-ß). All five switched sites, both conservative and radical, were localized within SBD-ß (fig. 5*B*; supplementary fig. S12, Supplementary Material online). Three of these five sites (P446, P449, and V466) are near or in the cleft itself (supplementary fig. S13, Supplementary Material online), and thus likely to affect the specificity of client binding (Zhu et al. 1996), a testable scenario that is consistent with these changes being important for functional specialization of *SSQ1*. Altogether, our data indicate that *SSQ1* was subjected to divergent evolution driven by lowered expression levels and functional diversification, but that critical function was maintained by purifying selection. This scenario is consistent with previously observed asymmetric rate of evolution between duplicate genes (Kim and Yi 2006), which is usually explained by differences in expression levels and relaxed purifying selection due to the partitioning of ancestral functions between duplicates.

As mentioned earlier, the phylogenetic distribution of the third SSC paralog, *SSC3*, is restricted to post-WGD species (fig. 1; supplementary fig. S4, Supplementary Material online). Evolution of *SSC3* varies in a taxon-specific manner. In two species (*C. glabrata and V. polyspora*) high sequence identity and branching pattern of the protein tree suggested a concerted mode of evolution driven most likely by gene conversion events. However, in the other species, *SSC3* diverged significantly from its parental gene. In fact, the d*N*/d*S* ratio determined for *SSC3* (table 2) was the highest among canonical Hsp70s. All fixed and switched sites are spatially distributed evenly along the linear *SSC3* sequence (fig. 5). Thus, in contrast to *SSQ1*, no specific region was indicative of functional divergence for *SSC3*. Even though the *S. cerevisiae SSC3* protein is associated with mitochondrial DNA (Sakasegawa et al. 2003), deletion of *SSC3* has no known phenotypic effects (Baumann et al. 2000; Pareek et al. 2011), including any functional relationship to mtDNA maintenance and propagation. However, *SSC3* is defective in its ability to interact with client peptides, likely due to its inability to perform ATPase-dependent allosteric interdomain communication, which may indicate it is early in the process of pseudogenization (Pareek et al. 2011). Yet, given that gene loss is rapid for nonfunctional gene in post-WGD species (Hittinger et al. 2004), the maintenance of *SSC3* orthologs in most post-WGD genomes strongly suggests a functional role. An intriguing possibility is that *SSC3* is emerging as a new atypical Hsp70 (Pareek et al. 2011). However, further experimental studies are needed to reveal whether *SSC3* should be classified as a pseudo, a canonical or, possibly, an atypical Hsp70.

### Atypical Hsp70s: Low Copy Number Dynamics and High Sequence Divergence

Copy number is relatively stable among atypical Hsp70s. In the 53 surveyed species, we identified 60 copies of SSE, 54 copies of SSZ, and 54 copies of LHS. Paralogous copies of SSE were found in 7 of the 8 *Saccharomycetaceae* analyzed and coincided with the WGD (fig. 1; supplementary fig. S5, Supplementary Material online); the 8th, *C. glabrata*, experienced a lineage-specific loss of one SSE copy. Paralogous copies of SSZ and LHS were found in a single species (*V. polyspora*) that branches at the base of *Saccharomycetaceae* post-WGD clade. This finding coupled with the results of synteny analysis implies that the additional copies of both LHS and SSZ present in *V. polyspora* originated during the WGD event. The lack of such SSZ and LHS paralogs in most post-WGD *Saccharomycetaceae* species, and therefore, likely resulted from multiple lineage- or species-specific loss events in other post-WGD species (fig. 1; supplementary figs. S6 and S7, Supplementary Material online).

Unlike the stability of copy number, atypical Hsp70s have high rates of sequence evolution, as indicated by the following: 1) long branches on the gene tree (fig. 1*B*; supplementary figs. S9–S11, Supplementary Material online), 2) relatively low mean pairwise amino acid identity (table 1), 3) high α values for amino acid usage, ranging from 1.21 for SSZ to 2.85 for LHS (fig. 3), and 4) d*N*/d*S* values that are markedly higher than those of most canonical Hsp70s (table 2). Thus, our data are consistent with atypical Hsp70s evolving under more relaxed purifying selection than canonical Hsp70s.

**Fig. 6.—:**
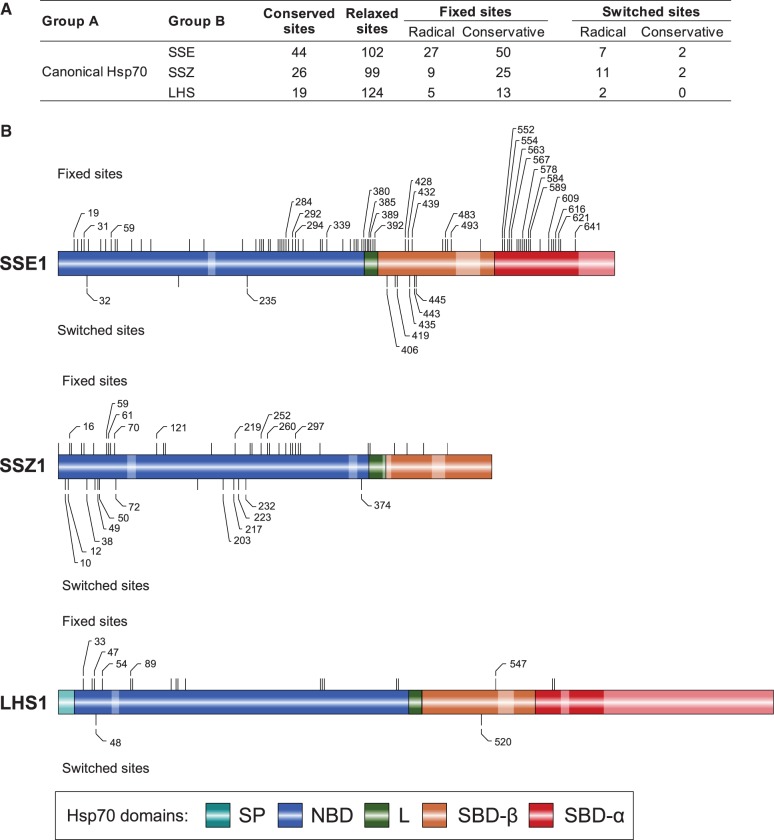
Amino acid sequence divergence of atypical SSE, SSZ, and LHS subfamilies. (*A*) Summary of sequence divergence analysis. Group A, canonical Hsp70 homologs (223 sequences) belonging to the SSA, KAR, and SSC subfamilies. Group B, Hsp70 orthologs belonging to the SSE (60 sequences), SSZ (54 sequences), and LHS (52 sequences) subfamilies, as indicated. Conserved sites—number of sites with residues identical in group A and B. Relaxed sites—number of sites that are invariant in A but variable in B. Fixed sites—number of sites that are variable in A but invariant in B. Switched sites—number of sites that are invariant within group A and group B but occupied by different amino acids in each group. Radical—number of sites where substitution from the most prevalent residue in group A to the most prevalent residue in group B had the BLOSUM62 score equal to or less than zero. Conservative—number of sites where substitution from the most prevalent residue in group A to the most prevalent residue in group B had the BLOSUM62 score strictly greater than zero. (*B*) Approximate localization of the radical fixed and radical switched sites within sequences orthologous to *SSE1*, *SSZ1*, and *LHS1*, as indicated. Fixed sites marked by bars above the sequence and switched sites marked by bars below sequence with radical sites marked by their sequence position. Pale fragments indicate patches of at least nine consecutive gaps in the alignment of sequences from groups A and B, and thus not subjected to the analysis. SP, signal peptide; L, Linker; SBD-α, substrate-binding domain alpha-helical lid.

To further characterize sequence divergence patterns of atypical Hsp70s, we analyzed the distribution of fixed and switched sites (fig. 6). Because of rapid sequence evolution among atypical Hsp70s, we compared sequences for each subfamily of atypical Hsp70s (termed Group B) against canonical Hsp70s (termed Group A) (fig. 6*A*). Consistent with high rates of sequence evolution, our divergence analysis revealed a very limited number of conserved sites across group A and group B alignments (fig. 6). 44 conserved sites were found for SSE, 26 for SSZ and only 19 such sites for LHS. Consistent with their high substitution rates, multiple fixed and switched sites were distributed evenly across the entire sequences of SSE and SSZ. Although only 18 fixed and 2 switched sites were detected for LHS, due to a very low level of sequence conservation. These sites exhibit no clear spatial pattern in their distribution across the sequence. During our analysis of sequence divergence of atypical Hsp70s, we noted another difference: subfamily-specific differences in their length due to specific insertions/deletions (fig. 6*B*).

Altogether, atypical Hsp70s evolve faster and subfamilies are more divergent than those of canonical Hsp70s. As fast evolving *SSE1* and *SSZ1* are expressed at high levels, comparable with that of most canonical Hsp70s, low expression is not a likely explanation for rapid evolution (table 2; figs. 3 and 6). The more likely explanation is relaxation of functional constraints. However, the situation is complex, because, while atypical Hsp70s are known to have a more limited ability to perform ATPase-dependent client binding and releasing cycle (Shaner and Morano 2007), they are also known to interact specifically with canonical Hsp70 partners (SSE and LHS) or with J-protein co-chaperones (SSZ). In addition, recent experimental studies reveals that their repertoire of biochemical activities is larger than expected based on earlier studies (Steel et al. 2004; Shaner and Morano 2007; Rampelt et al. 2012; Xu et al. 2012; Mattoo et al. 2013). However, despite recent findings, the limited allosteric communication between their two functional domains (NBD and SBD) (Liu and Hendrickson 2007; Schuermann et al. 2008) remains the most likely explanation for relaxed selection as the cause of higher substitution rates among atypical Hsp70s. Interdomain allosteric communication engages a large number of co-evolving residues, linking the functional sites of the two domains across a specific interdomain interface (Smock et al. 2010), and thus likely explaining lower substitution rates among canonical Hsp70s.

## Conclusions and Perspectives

The major finding of our analysis is that the Hsp70 chaperone family is highly conserved with members of the seven Hsp70 subfamilies, both canonical and atypical, present in all surveyed species. We postulate that each of these seven subfamilies diversified from their corresponding single ancestral gene that was present in the common ancestor of all surveyed species and which subsequently underwent both independent and genome-scale duplication events. As a result of independent duplicate gene retentions among subfamilies and taxa, we observed wide ranging gene family evolutionary dynamics. Yet, despite these rich evolution dynamics, we found no evidence for any changes in cellular localization or function across subfamilies. Thus, the Hsp70 subfamilies constitute conserved units of function and evolution. This relationship between subfamilies and functional units was first proposed based on the study of Hsp70 family in *S. cerevisiae* (Boorstein et al. 1994). Here, we demonstrate that this relationship is robust, supported after much greater taxon sampling and analysis of genomic sequence data sets. These findings may well be applicable to all eukaryotes, as data available for other taxonomic groups suggest a similar pattern, both in terms of evolutionary conservation of subfamilies and their consistent compartmental localization (Hughes 1993; Lin et al. 2001; Nikolaidis and Nei 2004; Wada et al. 2006; Daugaard et al. 2007; Georg Rde and Gomes 2007; Brocchieri et al. 2008; Kabani and Martineau 2008).

The mechanisms of Hsp70 gene family evolution varied among subfamilies and taxa. Some subfamilies evolved according to a simple divergent scenario with few changes in copy number dynamics (e.g., KAR, SSZ, and LHS). Others underwent highly dynamic changes in copy number and evolved according to the birth–death model of gene family evolution (SSA and SSC). In a few cases, subfamilies evolved under concerted evolution with gene conversion homogenizing amino acid sequences within the species (SSBs and to a more limited extent, SSA and SSC). Yet, a common theme emerges when our analysis is considered together with available functional data: within each Hsp70 subfamily paralogous genes diversified functionally regardless of their mode of evolution. In contrast to high copy number dynamics, the sequence evolution was rather conservative with purifying selection as the major evolutionary force. The rate of sequence evolution was inversely proportional to the expression level, as expected, as well as strongly functionally constrained. In general, atypical Hsp70s, which have limited allosteric communication between functional domains, evolved faster than canonical Hsp70s. Among canonicals, highly specialized and low abundant *SSQ1* evolved faster than multifunctional and highly abundant SSA, KAR and SSC; however, in all cases the rate of evolution for Hsp70s relative to the genome-wide average was very low, indicative of purifying selection as the major evolutionary force influencing sequence evolution.

Practically, how do our findings inform future studies? In the context of functional analyses, researchers using model organisms should consider that Hsp70 subfamilies, which constitute conserved functional units, experience complex evolutionary dynamics, often in a taxon-specific manner. As a consequence, functions associated with a given Hsp70 subfamily could be partitioned among a number of paralogous genes, whose homology relationships across species might be difficult to determine. Moreover, the manners in which these functions are distributed among paralogous genes may vary from model species to model species, so inference of function across species using only sequence homology should be made with caution. Yet, in some circumstances, it may be advantageous for experimentalists to exploit this situation, as it could allow study of a specific function associated with a specialized Hsp70 without interference from disruption of other activities typically carried out by multifunctional Hsp70s. For example, the presence of the specialized *SSQ1* gene in *S. cerevisiae*, has allowed detailed analysis of Hsp70s’ function in the mitochondrial biogenesis of iron–sulfur containing proteins, where genetic manipulation would have resulted in lethality in taxa without the duplicate gene (Craig and Marszalek 2011).

In the context of evolutionary studies, our results provide a striking example: even a highly conserved and ubiquitous protein family, whose members are present in all domains of life, performing universal and often essential functions is subjected to dynamic and variable evolutionary forces, which often act in a lineage-specific level. Yet, there is a common theme in these ostensibly chaotic evolutionary dynamics. The presence of each Hsp70 subfamily is conserved; thus, despite evolutionary complexity, subfamilies evolve in accordance to the protein family evolution model, wherein orthologous groups constitute units of function and evolution preserved across a phylogeny (Gabaldon and Koonin 2013). Finally, the evolutionary dynamics detected in our study are still difficult to interpret in functional terms. For example, why do taxa vary in copy number for the SSA subfamily? Why have mitochondrial Hsp70s specialized for iron–sulfur biogenesis (*SSQ1*) evolved only in a limited number of fungal species, given this specialization independently arose in the bacterial ancestors of mitochondria (Schilke et al. 2006)? Why do SSB paralogs independently maintain a few amino acid differences despite repeated homogenization across the rest of the protein sequence? How have interactions of Hsp70s with their co-chaperones, J-proteins and nucleotide release factors, affected patterns of evolution? It is clear that sequence analysis alone is not sufficient to answers these questions; only a combination of evolutionary analyses coupled with experimental studies will ultimately provide answers (Harms and Thornton 2013).

## Supplementary Material

Supplementary figures S1–S13 are available at *Genome Biology and Evolution* online (http://www.gbe.oxfordjournals.org/).

Supplementary Data

## References

[evt192-B1] Altschul SF, Gish W, Miller W, Myers EW, Lipman DJ (1990). Basic local alignment search tool. J Mol Biol..

[evt192-B2] Ayres DL (2012). BEAGLE: an application programming interface and high-performance computing library for statistical phylogenetics. Syst Biol..

[evt192-B3] Baumann F, Milisav I, Neupert W, Herrmann JM (2000). Ecm10, a novel hsp70 homolog in the mitochondrial matrix of the yeast *Saccharomyces cerevisiae*. FEBS Lett..

[evt192-B4] Bettencourt BR, Feder ME (2001). Hsp70 duplication in the *Drosophila melanogaster* species group: how and when did two become five?. Mol Biol Evol..

[evt192-B5] Bettencourt BR, Feder ME (2002). Rapid concerted evolution via gene conversion at the *Drosophila* hsp70 genes. J Mol Evol..

[evt192-B6] Boorstein WR, Ziegelhoffer T, Craig EA (1994). Molecular evolution of the HSP70 multigene family. J Mol Evol..

[evt192-B7] Breen MS, Kemena C, Vlasov PK, Notredame C, Kondrashov FA (2012). Epistasis as the primary factor in molecular evolution. Nature.

[evt192-B8] Broadley SA, Hartl FU (2009). The role of molecular chaperones in human misfolding diseases. FEBS Lett..

[evt192-B9] Brocchieri L, Conway de Macario E, Macario AJ (2008). hsp70 genes in the human genome: conservation and differentiation patterns predict a wide array of overlapping and specialized functions. BMC Evol Biol..

[evt192-B10] Brown CR, McCann JA, Chiang HL (2000). The heat shock protein Ssa2p is required for import of fructose-1, 6-bisphosphatase into Vid vesicles. J Cell Biol..

[evt192-B11] Byrne KP, Wolfe KH (2006). Visualizing syntenic relationships among the hemiascomycetes with the Yeast Gene Order Browser. Nucleic Acids Res..

[evt192-B12] Cascardo JC (2000). The phosphorylation state and expression of soybean BiP isoforms are differentially regulated following abiotic stresses. J Biol Chem..

[evt192-B13] Castresana J (2000). Selection of conserved blocks from multiple alignments for their use in phylogenetic analysis. Mol Biol Evol..

[evt192-B14] Clark MS, Fraser KPP, Peck LS (2008). Antarctic marine molluscs do have an HSP70 heat shock response. Cell Stress Chaperones.

[evt192-B15] Cock PJ (2009). Biopython: freely available Python tools for computational molecular biology and bioinformatics. Bioinformatics.

[evt192-B16] Craig EA, Marszalek J (2011). Hsp70 chaperones. Encyclopedia of life sciences (ELS).

[evt192-B17] Darriba D, Taboada GL, Doallo R, Posada D (2011). ProtTest 3: fast selection of best-fit models of protein evolution. Bioinformatics.

[evt192-B18] Daugaard M, Rohde M, Jaattela M (2007). The heat shock protein 70 family: highly homologous proteins with overlapping and distinct functions. FEBS Lett..

[evt192-B19] Denecke J, Goldman MHS, Demolder J, Seurinck J, Botterman J (1991). The tobacco luminal binding-protein is encoded by a multigene family. Plant Cell.

[evt192-B20] Deshaies RJ, Koch BD, Werner-Washburne M, Craig EA, Schekman R (1988). A subfamily of stress proteins facilitates translocation of secretory and mitochondrial precursor polypeptides. Nature.

[evt192-B21] Dover G (1982). Molecular drive: a cohesive mode of species evolution. Nature.

[evt192-B22] Drummond DA, Bloom JD, Adami C, Wilke CO, Arnold FH (2005). Why highly expressed proteins evolve slowly. Proc Natl Acad Sci U S A..

[evt192-B23] Dujon B (2010). Yeast evolutionary genomics. Nat Rev Genet..

[evt192-B24] Edgar RC (2004). MUSCLE: multiple sequence alignment with high accuracy and high throughput. Nucleic Acids Res..

[evt192-B25] Emanuelsson O, Brunak S, von Heijne G, Nielsen H (2007). Locating proteins in the cell using TargetP, SignalP and related tools. Nat Protoc..

[evt192-B26] Fitzpatrick DA, Logue ME, Stajich JE, Butler G (2006). A fungal phylogeny based on 42 complete genomes derived from supertree and combined gene analysis. BMC Evol Biol..

[evt192-B27] Fitzpatrick DA, O'Gaora P, Byrne KP, Butler G (2010). Analysis of gene evolution and metabolic pathways using the Candida Gene Order Browser. BMC Genomics.

[evt192-B28] Gabaldon T, Koonin EV (2013). Functional and evolutionary implications of gene orthology. Nat Rev Genet..

[evt192-B29] Georg Rde C, Gomes SL (2007). Comparative expression analysis of members of the Hsp70 family in the chytridiomycete *Blastocladiella emersonii*. Gene.

[evt192-B30] Germot A, Philippe H (1999). Critical analysis of eukaryotic phylogeny: a case study based on the HSP70 family. J Eukaryot Microbiol..

[evt192-B31] Gordon JL, Byrne KP, Wolfe KH (2009). Additions, losses, and rearrangements on the evolutionary route from a reconstructed ancestor to the modern *Saccharomyces cerevisiae* genome. PLoS Genet..

[evt192-B32] Gu X (1999). Statistical methods for testing functional divergence after gene duplication. Mol Biol Evol..

[evt192-B33] Gu X (2001). Maximum-likelihood approach for gene family evolution under functional divergence. Mol Biol Evol..

[evt192-B34] Guindon S, Gascuel O (2003). A simple, fast, and accurate algorithm to estimate large phylogenies by maximum likelihood. Syst Biol..

[evt192-B35] Gupta RS, Bustard K, Falah M, Singh D (1997). Sequencing of heat shock protein 70 (DnaK) homologs from *Deinococcus proteolyticus* and *Thermomicrobium roseum* and their integration in a protein-based phylogeny of prokaryotes. J Bacteriol..

[evt192-B36] Gupta RS, Singh B (1994). Phylogenetic analysis of 70 kD heat shock protein sequences suggests a chimeric origin for the eukaryotic cell nucleus. Curr Biol..

[evt192-B37] Harms MJ, Thornton JW (2013). Evolutionary biochemistry: revealing the historical and physical causes of protein properties. Nat Rev Genet..

[evt192-B38] He Y (2013). Identification of a testis-enriched heat shock protein and fourteen members of hsp70 family in the swamp eel. PLoS One.

[evt192-B39] Hittinger CT, Rokas A, Carroll SB (2004). Parallel inactivation of multiple GAL pathway genes and ecological diversification in yeasts. Proc Nati Acad Sci U S A..

[evt192-B40] Huang P, Gautschi M, Walter W, Rospert S, Craig EA (2005). The Hsp70 Ssz1 modulates the function of the ribosome-associated J-protein Zuo1. Nat Struct Mol Biol..

[evt192-B41] Hughes AL (1993). Nonlinear relationships among evolutionary rates identify regions of functional divergence in heat-shock protein 70 genes. Mol Biol Evol..

[evt192-B42] Jung KH, Gho HJ, Nguyen MX, Kim SR, An G (2013). Genome-wide expression analysis of HSP70 family genes in rice and identification of a cytosolic HSP70 gene highly induced under heat stress. Funct Integr Genomics..

[evt192-B43] Kabani M, Martineau CN (2008). Multiple hsp70 isoforms in the eukaryotic cytosol: mere redundancy or functional specificity?. Curr Genomics..

[evt192-B44] Kampinga HH, Craig EA (2010). The HSP70 chaperone machinery: J proteins as drivers of functional specificity. Nat Rev Mol Cell Biol..

[evt192-B45] Katoh K, Standley DM (2013). MAFFT multiple sequence alignment software version 7: improvements in performance and usability. Mol Biol Evol..

[evt192-B46] Kim SH, Yi SV (2006). Correlated asymmetry of sequence and functional divergence between duplicate proteins of *Saccharomyces cerevisiae*. Mol Biol Evol..

[evt192-B47] Kim YE, Hipp MS, Bracher A, Hayer-Hartl M, Hartl FU (2013). Molecular chaperone functions in protein folding and proteostasis. Annu Rev Biochem..

[evt192-B48] Kudla G, Helwak A, Lipinski L (2004). Gene conversion and GC-content evolution in mammalian Hsp70. Mol Biol Evol..

[evt192-B49] Le SQ, Gascuel O (2008). An improved general amino acid replacement matrix. Mol Biol Evol..

[evt192-B50] Lin BL (2001). Genomic analysis of the Hsp70 superfamily in *Arabidopsis thaliana*. Cell Stress Chaperones.

[evt192-B51] Liu Q, Hendrickson WA (2007). Insights into Hsp70 chaperone activity from a crystal structure of the yeast Hsp110 Sse1. Cell.

[evt192-B52] Louw CA, Ludewig MH, Mayer J, Blatch GL (2010). The Hsp70 chaperones of the Tritryps are characterized by unusual features and novel members. Parasitol Int..

[evt192-B53] Luan W, Li FH, Zhang JQ, Wang B, Xiang JH (2009). Cloning and expression of glucose regulated protein 78 (GRP78) in *Fenneropenaeus chinensis*. Mol Biol Rep..

[evt192-B54] MacIsaac KD (2006). An improved map of conserved regulatory sites for *Saccharomyces cerevisiae*. BMC Bioinformatics.

[evt192-B55] Maguire SL (2013). Comparative genome analysis and gene finding in *Candida* species using CGOB. Mol Biol Evol..

[evt192-B56] Marocco A (1991). Three high-lysine mutations control the level of ATP-binding HSP70-like proteins in the maize endosperm. Plant Cell.

[evt192-B57] Mattoo RU, Sharma SK, Priya S, Finka A, Goloubinoff P (2013). Hsp110 is a bona fide chaperone using ATP to unfold stable misfolded polypeptides and reciprocally collaborate with hsp70 to solubilize protein aggregates. J Biol Chem..

[evt192-B58] Mayer MP, Bukau B (2005). Hsp70 chaperones: cellular functions and molecular mechanism. Cell Mol Life Sci..

[evt192-B59] McClellan AJ (1998). Specific molecular chaperone interactions and an ATP-dependent conformational change are required during posttranslational protein translocation into the yeast ER. Mol Biol Cell..

[evt192-B60] Miskovic D, Salter-Cid L, Ohan N, Flajnik M, Heikkila JJ (1997). Isolation and characterization of a cDNA encoding a Xenopus immunoglobulin binding protein, BiP (grp78). Comp Biochem Physiol B Biochem Mol Biol..

[evt192-B61] Nei M, Rooney AP (2005). Concerted and birth-and-death evolution of multigene families. Annu Rev Genet..

[evt192-B62] Nikolaidis N, Nei M (2004). Concerted and nonconcerted evolution of the Hsp70 gene superfamily in two sibling species of nematodes. Mol Biol Evol..

[evt192-B63] Pareek G, Samaddar M, D'Silva P (2011). Primary sequence that determines the functional overlap between mitochondrial heat shock protein 70 Ssc1 and Ssc3 of *Saccharomyces cerevisiae*. J Biol Chem..

[evt192-B64] Peden J (1999). Analysis of codon usage.

[evt192-B65] Pfund C (1998). The molecular chaperone Ssb from *Saccharomyces cerevisiae* is a component of the ribosome-nascent chain complex. EMBO J..

[evt192-B96] R Development Core Team (2013). R: a language and environment for statistical computing [Internet] R version 3.0.1.

[evt192-B66] Rampelt H (2012). Metazoan Hsp70 machines use Hsp110 to power protein disaggregation. EMBO J..

[evt192-B67] Raviol H, Bukau B, Mayer MP (2006). Human and yeast Hsp110 chaperones exhibit functional differences. FEBS Lett..

[evt192-B68] Rhind N (2011). Comparative functional genomics of the fission yeasts. Science.

[evt192-B69] Ronquist F (2012). MrBayes 3.2: efficient Bayesian phylogenetic inference and model choice across a large model space. Syst Biol..

[evt192-B70] Sakasegawa Y, Hachiya NS, Tsukita S, Kaneko K (2003). Ecm10p localizes in yeast mitochondrial nucleoids and its overexpression induces extensive mitochondrial DNA aggregations. Biochem Biophys Res Commun..

[evt192-B71] Scannell DR (2011). The awesome power of yeast evolutionary genetics: new genome sequences and strain resources for the *Saccharomyces* sensu stricto genus. G3 (Bethesda).

[evt192-B72] Schilke B (2006). Evolution of mitochondrial chaperones utilized in Fe-S cluster biogenesis. Curr Biol..

[evt192-B73] Schuermann JP (2008). Structure of the Hsp110:Hsc70 nucleotide exchange machine. Mol Cell..

[evt192-B74] Shaner L, Morano KA (2007). All in the family: atypical Hsp70 chaperones are conserved modulators of Hsp70 activity. Cell Stress Chaperones.

[evt192-B75] Sharma D (2009). Function of SSA subfamily of Hsp70 within and across species varies widely in complementing *Saccharomyces cerevisiae* cell growth and prion propagation. PLoS One.

[evt192-B76] Sharma D, Masison DC (2011). Single methyl group determines prion propagation and protein degradation activities of yeast heat shock protein (Hsp)-70 chaperones Ssa1p and Ssa2p. Proc Nat Acad Sci U S A..

[evt192-B77] Sharp PM, Cowe E (1991). Synonymous codon usage in *Saccharomyces cerevisiae*. Yeast.

[evt192-B78] Smock RG (2010). An interdomain sector mediating allostery in Hsp70 molecular chaperones. Mol Syst Biol..

[evt192-B79] Stamatakis A (2006). RAxML-VI-HPC: maximum likelihood-based phylogenetic analyses with thousands of taxa and mixed models. Bioinformatics.

[evt192-B80] Steel GJ, Fullerton DM, Tyson JR, Stirling CJ (2004). Coordinated activation of Hsp70 chaperones. Science.

[evt192-B81] Sugino RP, Innan H (2006). Selection for more of the same product as a force to enhance concerted evolution of duplicated genes. Trends Genet..

[evt192-B82] Takuno S, Innan H (2009). Selection to maintain paralogous amino acid differences under the pressure of gene conversion in the heat-shock protein genes in yeast. Mol Biol Evol..

[evt192-B83] Taylor JW, Berbee ML (2006). Dating divergences in the fungal tree of life: review and new analyses. Mycologia.

[evt192-B84] Thomas-Chollier M (2008). RSAT: regulatory sequence analysis tools. Nucleic Acids Res..

[evt192-B85] Wada S, Hamada M, Satoh N (2006). A genomewide analysis of genes for the heat shock protein 70 chaperone system in the ascidian *Ciona intestinalis*. Cell Stress Chaperones.

[evt192-B86] Wang M (2012). PaxDb, a database of protein abundance averages across all three domains of life. Mol Cell Proteomics.

[evt192-B87] Werner-Washburne M, Becker J, Kosic-Smithers J, Craig EA (1989). Yeast Hsp70 RNA levels vary in response to the physiological status of the cell. J Bacteriol..

[evt192-B88] Werner-Washburne M, Stone DE, Craig EA (1987). Complex interactions among members of an essential subfamily of hsp70 genes in *Saccharomyces cerevisiae*. Mol Cell Biol..

[evt192-B89] Wolfe KH, Shields DC (1997). Molecular evidence for an ancient duplication of the entire yeast genome. Nature.

[evt192-B90] Wright F (1990). The ‘effective number of codons’ used in a gene. Gene.

[evt192-B91] Xi XZ, Ma KS (2013). Molecular cloning and expression analysis of glucose-regulated protein 78 (GRP78) gene in silkworm *Bombyx mori*. Biologia.

[evt192-B92] Xu X (2012). Unique peptide substrate binding properties of 110-kDa heat-shock protein (Hsp110) determine its distinct chaperone activity. J Biol Chem..

[evt192-B93] Xuereb B (2012). Molecular characterization and mRNA expression of grp78 and hsp90A in the estuarine copepod *Eurytemora affinis*. Cell Stress Chaperones.

[evt192-B94] Yang Z (2007). PAML 4: phylogenetic analysis by maximum likelihood. Mol Biol Evol..

[evt192-B95] Zhu X (1996). Structural analysis of substrate binding by the molecular chaperone DnaK. Science.

