# Supplementary material for: The Complex Evolutionary Dynamics of Hsp70s: A Genomic and Functional Perspective
Source: Genome Biol Evol. 2013 Nov 24;5(12):2460–77. doi: 10.1093/gbe/evt192 (PMC3879978; doi:10.1093/gbe/evt192)
Supplement: Supplementary Data [file supp_5_12_2460__index.html]

The Complex Evolutionary Dynamics of Hsp70s: A Genomic and Functional Perspective — The Complex Evolutionary Dynamics of Hsp70s: A Genomic and Functional Perspective — Supplementary Data 

# The Complex Evolutionary Dynamics of Hsp70s: A Genomic and Functional Perspective

## Supplementary Data

files

**Files in this Data Supplement:**

- Supplementary Data - pdf file
- Supplementary Data - xls file
