# Supplementary material for: The Complex Evolutionary Dynamics of Hsp70s: A Genomic and Functional Perspective
Source: Genome Biol Evol. 2013 Nov 24;5(12):2460–77. doi: 10.1093/gbe/evt192 (PMC3879978; doi:10.1093/gbe/evt192)

Supplementary Figures

Fig. S2.
(A) Bayesian tree of amino acid sequences from the SSB subfamily. The tree was rooted using SSB orthologs from Basidiomycota. Scale is in expected amino acid substitutions per site. (**) - posterior probability ≥ 0.95. **(B)** Bayesian tree of nucleotide sequences of SSB ORFs from post-WGD Saccharomycetaceae species. Scale is in expected nucleotide substitutions per site.

Fig. S3.
Bayesian tree of amino acid sequences from the KAR subfamily. The tree was rooted using KAR orthologs from Basidiomycota. Scale is in expected amino acid substitutions per site. (**) - posterior probability ≥ 0.95.

Fig. S4
(A) Bayesian tree of amino acid sequences from the SSC subfamily. The tree was rooted using SSC orthologs from Basidiomycota. Scale is in expected amino acid substitutions per site. (**) - posterior probability ≥ 0.95. $SSC1_{preSSQ1}$ - *SSC1* sequences from species that pre-date the emergence of *SSQ1*; $SSC1_{preSSC3}$ - *SSC1* sequences from Saccharomycetaceae pre-WGD species that pre-date the emergence of *SSC3*. (B) Cladogram showing the hypothetical scenario of SSC evolution in the CTG and Saccharomycetaceae clades. Arrows indicate genes evolving under concerted evolution.

Fig. S5.
(A) Bayesian tree of amino acid sequences from the SSE subfamily. The tree was rooted using SSE orthologs from Basidiomycota. Scale is in expected amino acid substitutions per site. (**) - posterior probability ≥ 0.95. (B) Cladogram showing the hypothetical scenario of SSE evolution in the Saccharomycetaceae clade.

Fig. S6.
(A) Bayesian tree of amino acid sequences from the SSZ subfamily. The tree was rooted using SSZ orthologs from Basidiomycota. Scale is in expected amino acid substitutions per site. (**) - posterior probability ≥ 0.95. (B) Cladogram showing the hypothetical scenario of SSZ evolution in the Saccharomycetaceae clade.

Fig. S7.
(A) Bayesian tree of amino acid sequences from the LHS subfamily. The tree was rooted using LHS orthologs from Basidiomycota. Scale is in expected amino acid substitutions per site. (**) - posterior probability ≥ 0.95. (B) Cladogram showing the hypothetical scenario of LHS evolution in the Saccharomycetaceae clade.

Fig. S8.
Bayesian tree of amino acid sequences from the SSA subfamily in the Yarrowia clade. The tree was rooted using *SSA1* from *S. cerevisiae*. Scale is in expected amino acid substitutions per site. Posterior probabilities are shown for the individual branches.

Fig. S9.
Amino acid usage for Hsp70 subfamilies defined as number of different amino acids observed at a site in the sequence alignment (see Materials and Methods for details).

Fig. S10.
P-values of the Kolmogorov-Smirnov tests comparing codon usage and evolutionary rates of the 14 Hsp70 orthologs in 5 Saccharomyces sensu-stricto species: *S. cerevisiae, S. paradoxus, S. mikatae, S. kudriavzevii* and *S. uvarum*. Values below 0.05 are marked in bold. (A) Codon usage comparisons: values below the main diagonal correspond to CAI comparisons, values above the main diagonal correspond to ENC comparisons. (B) Evolutionary rate comparisons: values below the main diagonal correspond to dN comparisons, values above the main diagonal correspond to dS comparisons.

Fig. S11.
List of transcription factors whose binding sites were analyzed for 5'IGS of *SSB1/SSB2* paralogs. In bold TF sites not present in species pre-dating SSB gene duplication.

Fig. S12.
List of switched sites from fig. 5 and fig. 6. Group A and group B – Hsp70 subfamilies as in fig. 5 and fig. 6. Positions are indicated as in the *S. cerevisiae* ortholog. Dominant residue – residue most frequently present at a given site. BLOSUM62 score – substitution score between the dominant residue from Group A and dominant residue from Group B. Radical switched sites in bold.

Fig. S13.
Localization of the switched radical sites on the structural model of the SBD of *SSQ1* from *S. cerevisaie*, which was generated by homology modeling using SWISS-MODEL (Arnold et al. 2006) and structure of DnaK (PDB: 2KHO) as template. The SBD-β domain is shown in orange, the SBD-α domain is shown in red. In green are the switched radical sites at indicated positions. In blue are sites homologous to DnaK sites known to participate in substrate binding (Zhu et al. 1996).

Arnold K, Bordoli L, Kopp J, Schwede T. 2006. The SWISS-MODEL workspace: a web-based environment for protein structure homology modelling. Bioinformatics 22: 195-201.

Zhu X, Zhao X, Burkholder WF, Gragerov A, Ogata CM, Gottesman ME, Hendrickson WA. 1996. Structural analysis of substrate binding by the molecular chaperone DnaK. Science 272: 1606-1614.

Supplementary Figure S2

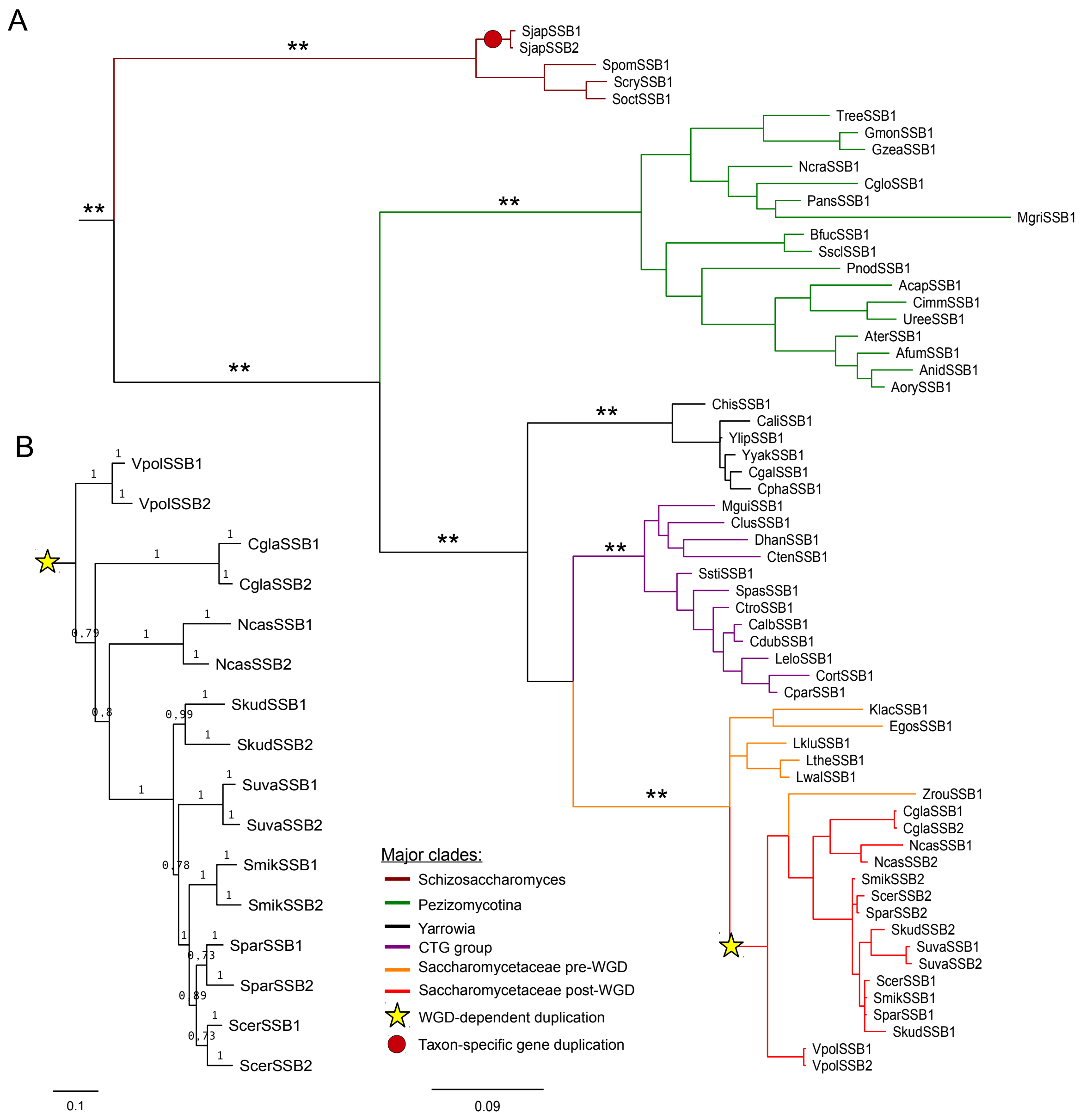

Supplementary Figure S3

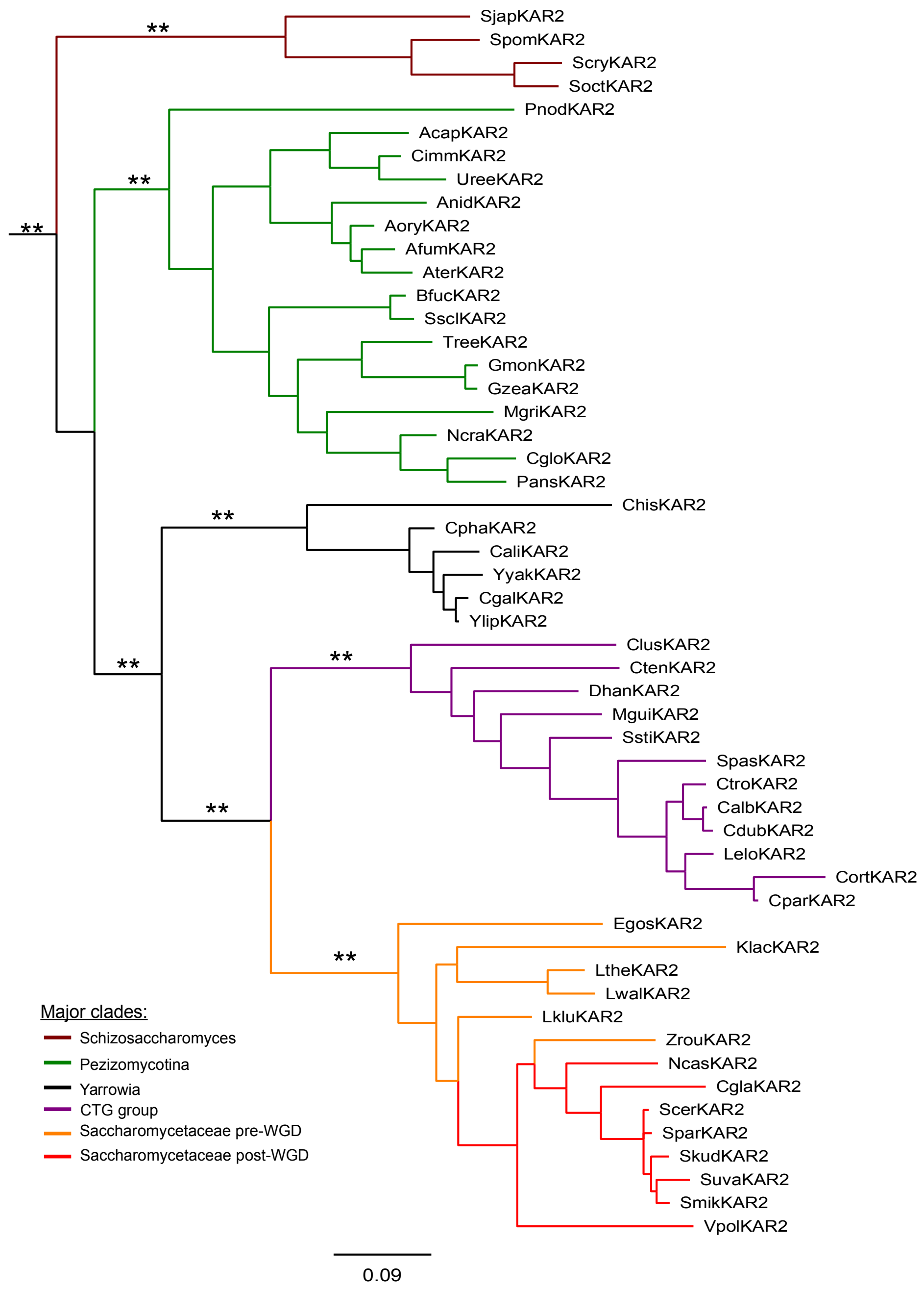

Supplementary Figure S4

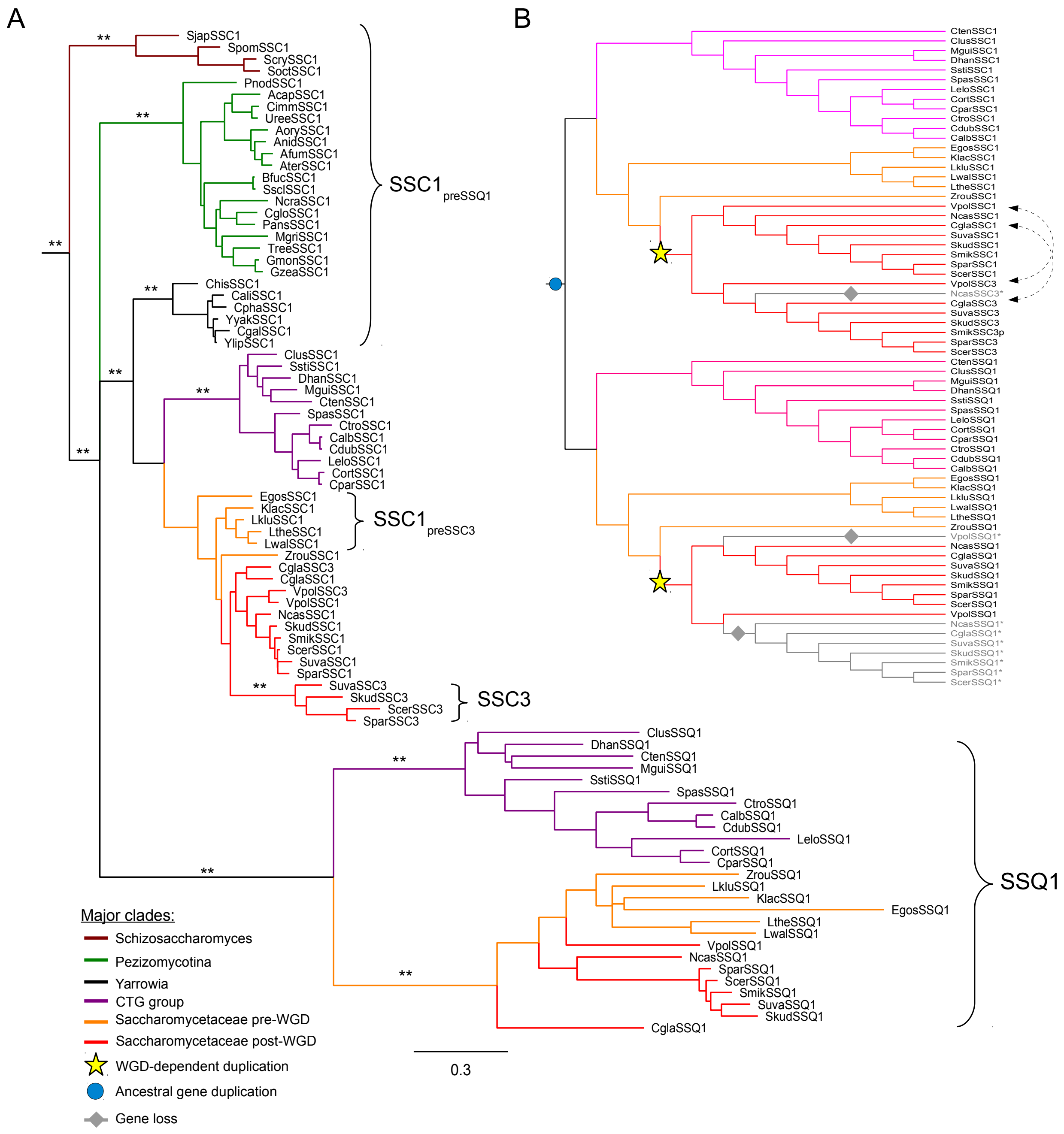

Supplementary Figure S5

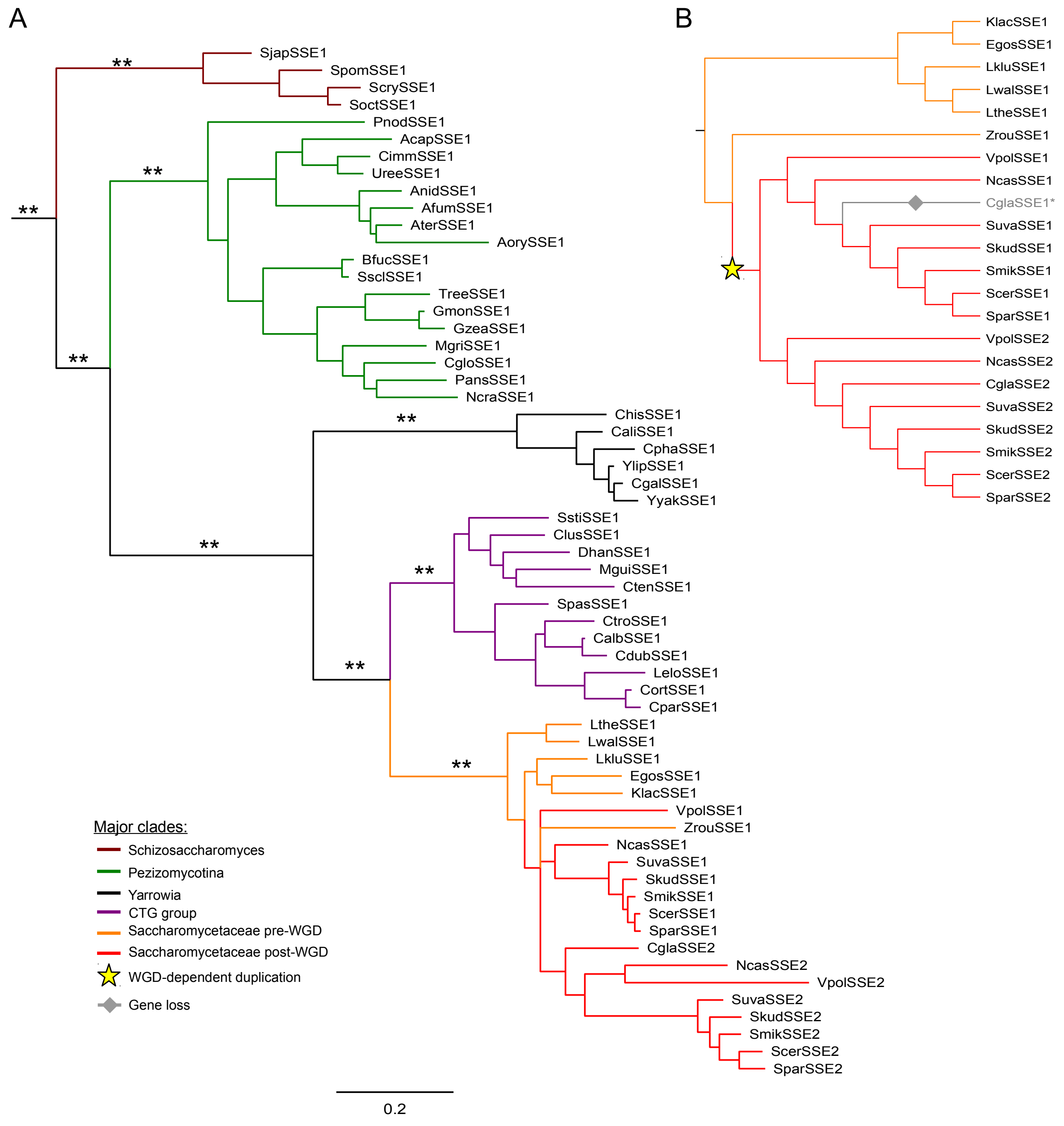

Supplementary Figure S6

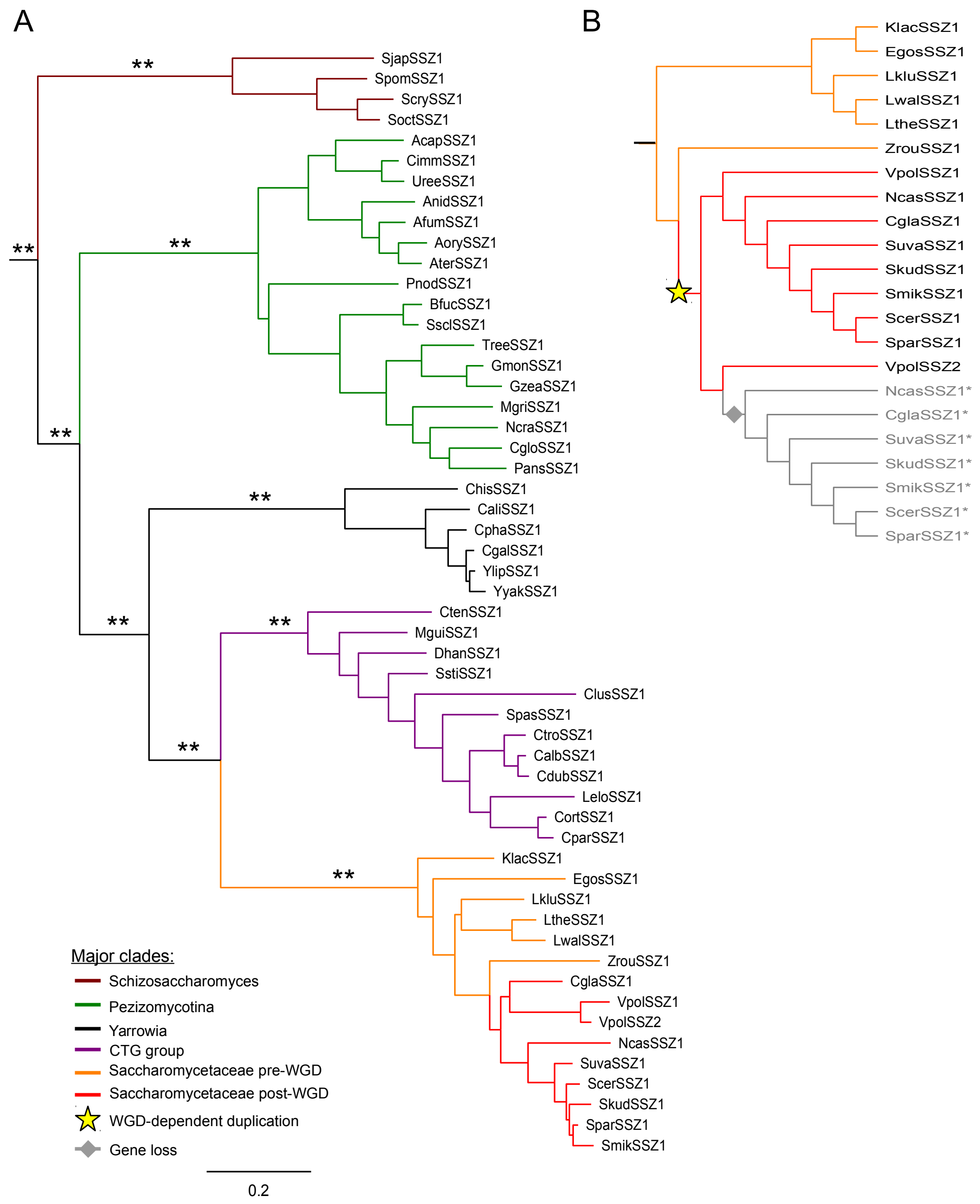

Supplementary Figure S7

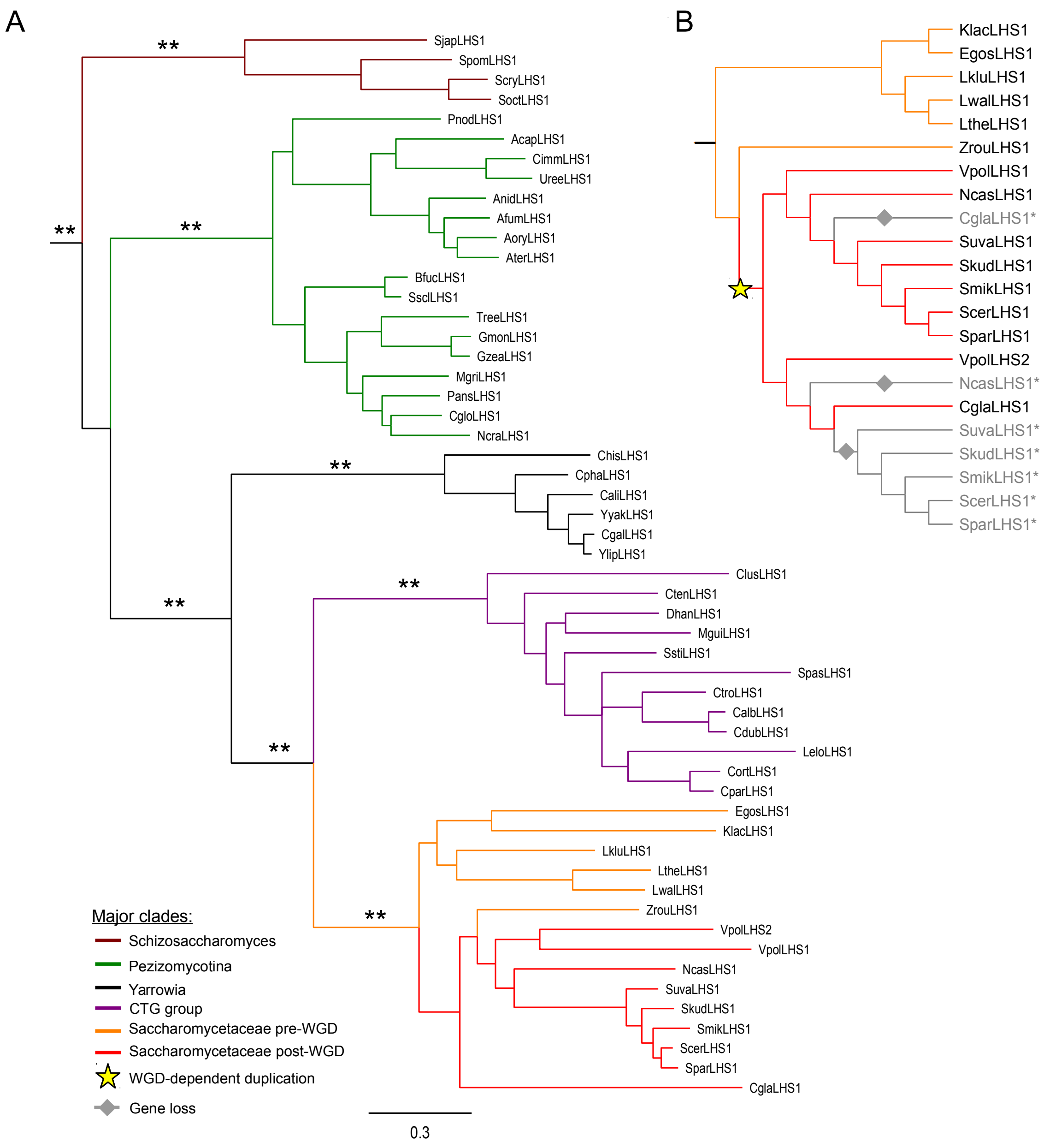

Supplementary Figure S8

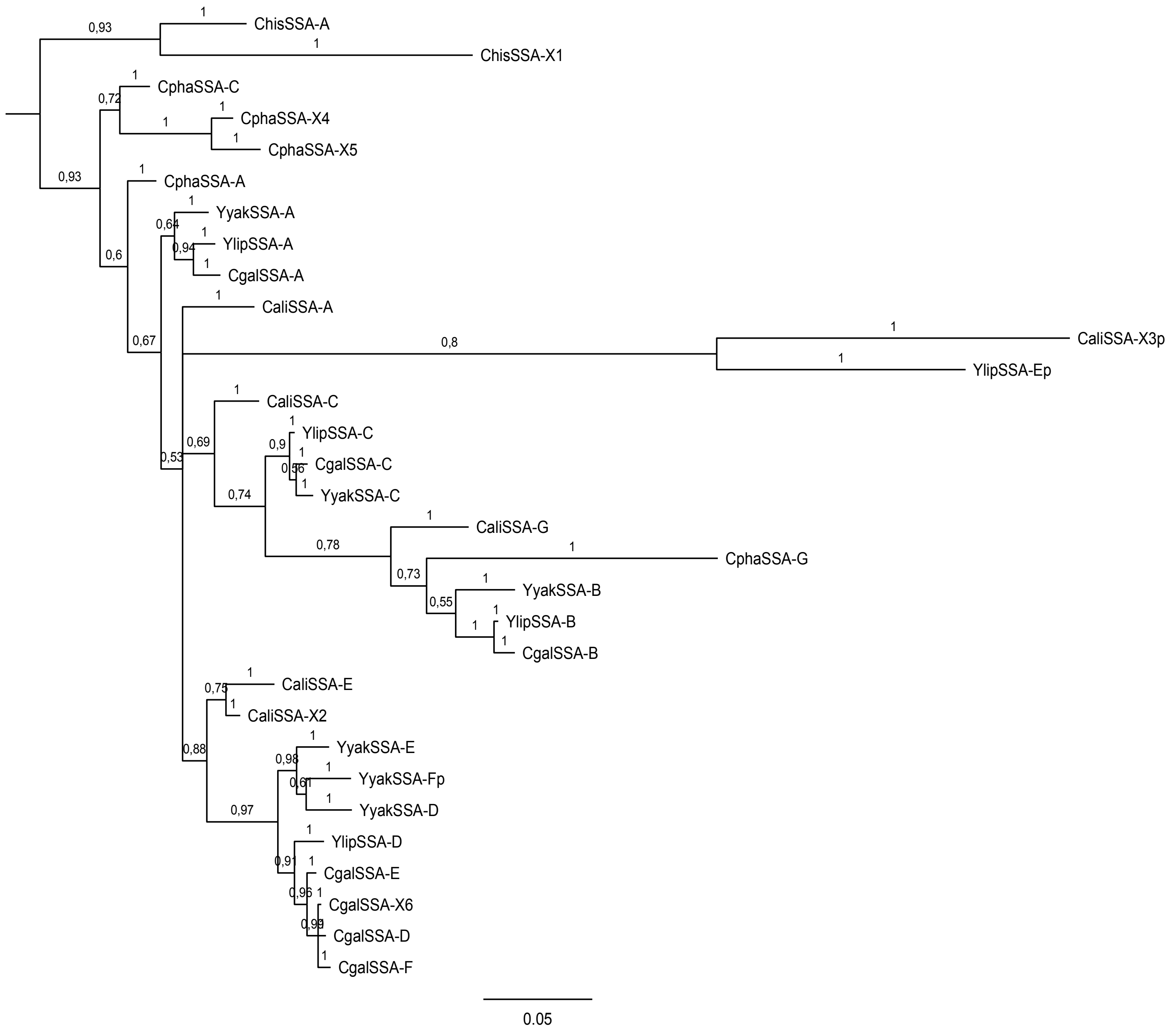

Supplementary Figure S9

Amino-acid usage	Number of sites							
	SSA	SSB	SSC	KAR	SSQ	SSE	SSZ	LHS
1	317	286	292	243	196	108	47	31
2	126	153	142	134	97	111	89	31
3	60	74	70	92	78	116	74	44
4	38	38	46	48	60	71	70	64
5	21	25	22	36	52	64	60	48
6	12	20	27	24	37	56	52	51
7	14	6	16	24	32	36	37	57
8	13	5	11	16	23	31	26	50
9	9	1	4	8	13	15	25	71
10	2	0	0	4	10	15	13	78
11	1	0	0	0	3	12	12	57
12	0	0	0	2	3	3	5	41
13	1	0	0	0	0	0	0	38
14	0	0	0	0	0	0	1	17
15	0	0	0	0	0	0	0	7
16	0	0	0	0	0	0	0	7
TOTAL	614	608	630	631	604	638	511	692

Supplementary Figure S10

A

**CAI \ ENC**	SSA1	SSA2	SSA3	SSA4	KAR2	SSB1	SSB2	SSC1	SSC3	SSQ1	SSE1	SSE2	SSZ1	LHS1
SSA1	-	***0.008***	***0.008***	***0.008***	***0.008***	***0.008***	***0.013***	***0.008***	***0.016***	***0.013***	0.357	***0.008***	***0.008***	***0.013***
SSA2	***0.013***	-	***0.008***	***0.008***	***0.008***	0.873	0.819	***0.008***	***0.016***	***0.013***	***0.008***	***0.008***	***0.008***	***0.013***
SSA3	***0.008***	***0.013***	-	0.873	***0.008***	***0.008***	***0.013***	***0.008***	0.429	***0.013***	***0.008***	0.873	***0.008***	***0.013***
SSA4	***0.008***	***0.013***	0.079	-	***0.008***	***0.008***	***0.013***	***0.008***	0.746	***0.013***	***0.008***	0.873	***0.008***	***0.013***
KAR2	***0.008***	***0.013***	***0.008***	***0.008***	-	***0.008***	***0.013***	***0.008***	***0.016***	***0.013***	***0.008***	***0.008***	1.000	***0.013***
SSB1	***0.008***	0.819	***0.008***	***0.008***	***0.008***	-	0.329	***0.008***	***0.016***	***0.013***	***0.008***	***0.008***	***0.008***	***0.013***
SSB2	***0.008***	0.329	***0.008***	***0.008***	***0.008***	0.873	-	***0.013***	***0.023***	***0.013***	***0.013***	***0.013***	***0.013***	***0.013***
SSC1	***0.008***	***0.013***	***0.008***	***0.008***	0.873	***0.008***	***0.008***	-	***0.016***	***0.013***	0.329	***0.008***	0.079	***0.013***
SSC3	***0.016***	***0.023***	0.286	***0.016***	***0.016***	***0.016***	***0.016***	***0.016***	-	***0.023***	***0.016***	0.869	***0.016***	***0.023***
SSQ1	***0.008***	***0.013***	0.357	***0.008***	***0.008***	***0.008***	***0.008***	***0.008***	0.079	-	***0.013***	***0.013***	***0.013***	0.819
SSE1	0.079	***0.013***	***0.008***	***0.008***	0.079	***0.008***	***0.008***	0.819	***0.016***	***0.008***	-	***0.008***	0.079	***0.013***
SSE2	***0.008***	***0.013***	0.357	***0.008***	***0.008***	***0.008***	***0.008***	***0.008***	0.286	0.873	***0.008***	-	***0.008***	***0.013***
SSZ1	***0.008***	***0.013***	***0.008***	***0.008***	0.873	***0.008***	***0.008***	0.357	***0.016***	***0.008***	0.357	***0.008***	-	***0.013***
LHS1	***0.013***	***0.013***	0.329	***0.013***	***0.013***	***0.013***	***0.013***	***0.013***	0.116	0.329	***0.013***	0.329	***0.013***	-

B

**dN \ dS**	SSA1	SSA2	SSA3	SSA4	KAR2	SSB1	SSB2	SSC1	SSC3	SSQ1	SSE1	SSE2	SSZ1	LHS1
SSA1	-	0.575	0.575	0.575	0.963	0.575	0.575	0.963	0.737	0.575	0.963	0.575	0.963	0.963
SSA2	0.541	-	0.963	0.963	0.963	0.575	0.963	0.963	0.116	0.575	0.963	0.575	0.575	0.963
SSA3	0.212	***0.008***	-	0.963	0.575	0.575	0.575	0.575	0.639	0.963	0.963	0.963	0.575	1.000
SSA4	0.212	0.203	0.938	-	0.575	0.212	0.575	0.575	0.737	0.963	0.963	0.963	0.575	1.000
KAR2	0.938	0.203	0.541	0.541	-	0.963	0.963	0.963	0.328	0.575	0.575	0.575	0.963	0.575
SSB1	0.575	0.575	***0.008***	0.053	0.203	-	0.963	0.963	***0.030***	0.575	0.575	0.212	0.575	0.575
SSB2	0.575	1.000	***0.008***	0.053	0.203	0.938	-	0.938	***0.030***	0.212	0.575	0.212	0.575	0.575
SSC1	0.963	0.575	0.541	0.575	1.000	0.203	0.212	-	0.116	0.575	0.963	0.575	0.963	0.575
SSC3	***0.003***	***0.003***	***0.030***	***0.015***	***0.006***	***0.003***	***0.003***	***0.015***	-	0.737	0.116	0.576	0.545	0.737
SSQ1	***0.001***	***0.001***	0.212	0.053	***0.012***	***0.001***	***0.001***	***0.008***	***0.030***	-	0.963	0.963	0.575	0.963
SSE1	0.212	0.056	0.963	0.963	0.541	***0.008***	***0.008***	0.575	***0.015***	0.053	-	0.575	0.575	0.963
SSE2	***0.001***	***0.001***	0.212	0.053	***0.002***	***0.001***	***0.001***	***0.012***	0.116	0.575	0.203	-	0.575	0.938
SSZ1	0.212	***0.008***	0.541	0.541	0.203	***0.008***	***0.008***	0.575	***0.030***	0.575	0.963	0.575	-	0.575
LHS1	***0.001***	***0.001***	***0.001***	***0.001***	***0.002***	***0.001***	***0.001***	***0.001***	0.545	0.056	***0.001***	0.212	***0.008***	-

Supplementary Figure S11

TF	Presence	Description
Abf1	SSB1 and SSB2	DNA binding protein with possible chromatin-reorganizing activity involved in transcriptional activation, gene silencing, and DNA replication and repair.
Ace2	SSB1 and SSB2	Transcription factor required for septum destruction after cytokinesis.
Pho2	SSB1 and SSB2	Homeobox transcription factor; regulatory targets include genes involved in phosphate metabolism.
Rme1	SSB1 and SSB2	Zinc finger protein involved in control of meiosis.
Spt23	SSB1 and SSB2	ER membrane protein involved in regulation of OLE1 transcription, acts with homolog Mga2p.
Ste12	SSB1 and SSB2	Transcription factor that is activated by a MAP kinase signaling cascade, activates genes involved in mating or pseudohyphal/invasive growth pathways.
Mac1	SSB1	Copper-sensing transcription factor involved in regulation of genes required for high affinity copper transport.
Rap1	SSB1	Essential DNA-binding transcription regulator that binds at many loci.
**Rfx1**	**SSB1**	**Major transcriptional repressor of DNA-damage-regulated genes, recruits repressors Tup1p and Cyc8p to their promoters.**
Sfp1	SSB1	Regulates transcription of ribosomal protein and biogenesis genes.
Yap5	SSB1	Basic leucine zipper (bZIP) iron-sensing transcription factor.
Yhp1	SSB1	Homeobox transcriptional repressor.
Dal80	SSB2	Negative regulator of genes in multiple nitrogen degradation pathways.
**Gln3**	**SSB2**	**Transcriptional activator of genes regulated by nitrogen catabolite repression (NCR), localization and activity regulated by quality of nitrogen source.**
Hap3/Hap5	SSB2	Subunit of the heme-activated, glucose-repressed Hap2p/3p/4p/5p CCAAT-binding complex, a transcriptional activator and global regulator of respiratory gene expression.
Opi1	SSB2	Transcriptional regulator of a variety of genes; phosphorylation by protein kinase A stimulates Opi1p function in negative regulation of phospholipid biosynthetic genes.

Supplementary Figure S12

Group A	Group B	Switched Position	Dominant residue		BLOSUM62 score	Domain
			Group A	Group B		
SSC1	SSQ1	**446**	**R**	**P**	**-2**	**SBD-α**
		**449**	**N**	**P**	**-2**	**SBD-α**
		454	I	V	3	SBD-α
		**466**	**A**	**V**	**0**	**SBD-α**
		**558**	**Y**	**N**	**-2**	**SBD-β**
SSC1	SSC3	83	A	S	1	NBD
		85	V	I	3	NBD
		116	Y	F	3	NBD
		174	S	A	1	NBD
		207	E	D	2	NBD
		**325**	**F**	**L**	**0**	**NBD**
		**330**	**E**	**A**	**-1**	**NBD**
		333	V	I	3	NBD
		358	I	L	2	NBD
		380	E	D	2	NBD
		**381**	**P**	**A**	**-1**	**NBD**
		393	I	L	2	NBD
		403	A	S	1	NBD
		436	T	S	1	SBD-α
		458	I	V	3	SBD-α
		469	R	K	2	SBD-α
		501	D	N	1	SBD-α
		509	R	K	2	SBD-α
		517	A	S	1	SBD-α
		524	S	A	1	SBD-α
		538	D	E	2	SBD-α
		581	E	D	2	SBD-β
		582	A	S	1	SBD-β
		585	V	L	1	SBD-β
		603	E	D	2	SBD-β
		**614**	**T**	**I**	**-1**	**SBD-β**
Canonical Hsp70	SSE	**32**	**G**	**S**	**0**	**NBD**
		148	Y	W	2	NBD
		**235**	**E**	**R**	**0**	**NBD**
		**406**	**I**	**W**	**-3**	**SBD-α**
		418	L	V	1	SBD-α
		**419**	**I**	**F**	**0**	**SBD-α**
		**435**	**T**	**R**	**-1**	**SBD-α**
		**443**	**I**	**A**	**-1**	**SBD-α**
		**445**	**V**	**Y**	**-1**	**SBD-α**
Canonical Hsp70	SSZ	**10**	**L**	**F**	**0**	**NBD**
		**12**	**T**	**N**	**0**	**NBD**
		**38**	**T**	**I**	**-1**	**NBD**
		44	F	Y	3	NBD
		**49**	**R**	**E**	**0**	**NBD**
		**50**	**L**	**Y**	**-1**	**NBD**
		**72**	**R**	**D**	**-2**	**NBD**
		170	R	Q	1	NBD
		**203**	**T**	**R**	**-1**	**NBD**
		**217**	**E**	**T**	**-1**	**NBD**
		**223**	**G**	**H**	**-2**	**NBD**
		**232**	**F**	**L**	**0**	**NBD**
		**374**	**A**	**L**	**-1**	**NBD**
Canonical Hsp70	LHS	**46**	**N**	**T**	**0**	**NBD**
		**520**	**A**	**C**	**0**	**SBD-α**

Supplementary Figure S13

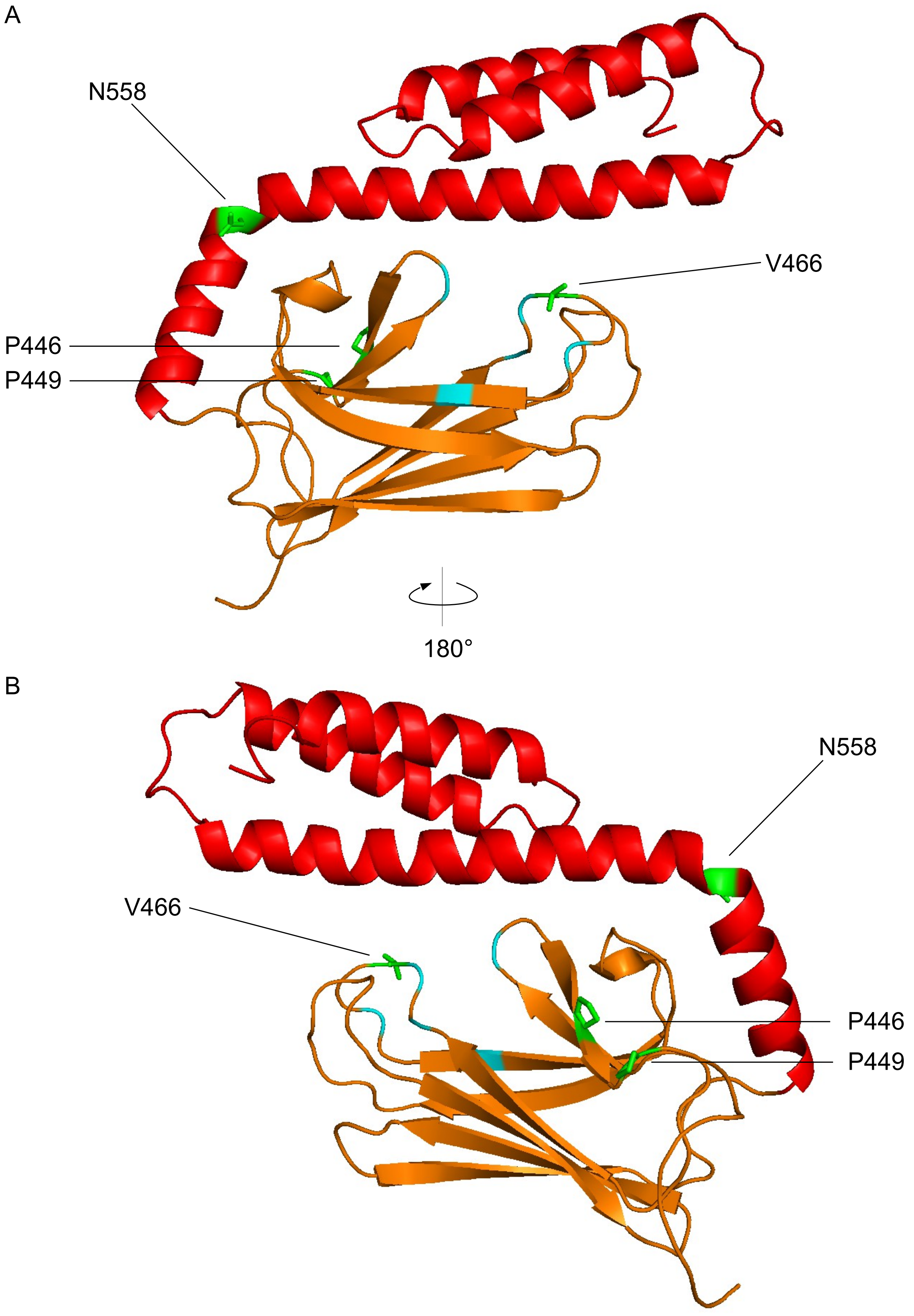

Supplement: Supplementary Data [file supp_evt192_Kominek_sup_figs_GBE.pdf]
